# Evaluation of the efflux inhibitory potential of gallotannin to restore drug sensitivity in XDR *Acinetobacter baumannii in vitro*, and a zebrafish infection model

**DOI:** 10.3389/fphar.2026.1652777

**Published:** 2026-04-08

**Authors:** Ramaravinth Manivannan, Niraimathi Muralidharan, Sadhiskumar Balavarun, Y. B. R. D. Rajesh, Aravind Sivasubramanian, Subramanian Muthukumar, Saisubramanian Nagarajan

**Affiliations:** 1 School of Chemical and Biotechnology, SASTRA Deemed University, Thanjavur, India; 2 Center for Research in Infectious Diseases, SASTRA Deemed University, Thanjavur, India; 3 Department of Chemistry, School of Chemical and Biotechnology, SASTRA Deemed University, Thanjavur, India

**Keywords:** *Acinetobacter baumannii*, Multidrug resistance (MDR/XDR), efflux pump inhibition, gallotannin, AdeB transporter, antibiotic potentiation, Reactive oxygen species (ROS), zebrafish infection model

## Abstract

**Introduction:**

*Acinetobacter baumannii* is a highly problematic nosocomial pathogen, designated a “red alert” organism by the Infectious Diseases Society of America. Multidrug-resistant (MDR) strains are associated with mortality rates of 25–68%, particularly in critically ill patients. Efflux-mediated resistance, along with serum-induced upregulation of efflux pump genes, highlights the role of efflux systems in driving MDR phenotypes. Targeting these systems represents a promising therapeutic strategy.

**Methods:**

The efflux inhibitory potential of 13 plant-derived polyphenols was evaluated to restore antibiotic susceptibility in MDR/XDR clinical isolates of *A. baumannii*. Approaches included *in silico* molecular docking and molecular dynamics simulations, *in vitro* assays (MIC reversal, real-time efflux, checkerboard synergy, intracellular accumulation, membrane permeability, and ROS generation), and *in vivo* bioburden analysis using a zebrafish infection model.

**Results and Discussion:**

Gallotannin exhibited the strongest activity, achieving a 64-fold reduction in erythromycin MIC in an XDR strain. Efflux inhibition was confirmed through real-time efflux and accumulation assays, showing 32–64-fold MIC reductions across multiple antibiotic classes in five MDR/XDR isolates. Molecular simulations revealed stable binding of gallotannin to the AdeB efflux pump via persistent hydrogen bonding. Combination therapy resulted in a ∼5-log CFU reduction *in vitro* and ∼4-log reduction in zebrafish bioburden. Mechanistically, gallotannin enhanced membrane permeability and intracellular antibiotic accumulation, without affecting membrane potential. The combination also induced significantly elevated ROS levels.Gallotannin potentiates erythromycin activity by inhibiting efflux, increasing permeability, and promoting ROS-mediated killing, significantly reducing A. baumannii burden *in vitro* and *in vivo*.

## Introduction

*Acinetobacter baumannii* is a Gram-negative coccobacillus, a ubiquitous nosocomial pathogen that causes a range of infections including wound, respiratory, UTI, and sepsis. It is one of the most predominant causes of neonatal sepsis as revealed by a large-scale study in India. In addition, 82% of *Acinetobacter spp.* isolated from neonates with sepsis were multidrug resistant (Agarwal et al., 2016). *A. baumannii* is most difficult to treat as it demonstrates high resilience, enhanced dissemination, and greater genome plasticity (Zhang et al., 2018). Due to its natural competence to acquire foreign DNA, *A. baumannii* typically acquires a drug-resistant phenotype. Among the multidrug resistant (MDR) *A. baumannii* isolates, carbapenem-resistant *A. baumannii* (CRAB) has been classified as a critical priority pathogen by WHO (Tacconelli et al., 2018), requiring urgent attention to develop new/effective therapies. The frequency of CRAB isolates ranges from 15% to 90% across the globe (Nguyen and Joshi, 2021; Nguyen and Joshi, 2021).

Although reasons such as reduced permeability, target site mutations, beta-lactamases, and drug modification have been attributed to the increased prevalence of drug-resistant strains, drug efflux plays a predominant role in conferring resistance to antimicrobials (Krishnamoorthy et al., 2017). *A. baumannii* possesses a wide range of efflux transporters belonging to all classes: RND pumps (AdeABC, AdeFGH, AdeIJK, AdeXYZ, AcrAB, etc.), MATE pumps (AbeM), Smr pumps (AbeS and QacE), MFS pumps (TetA, CraA, AmvA, EmrAB, AbaQ, etc.), ABC pumps (MacABTolC), and PACE pumps (AceI) (Zack et al., 2024). In addition to well-characterized efflux transporters, the genome of *A. baumannii* harbors multiple putative efflux pumps which have been shown to be differentially expressed under physiologically relevant salt conditions (Hood et al., 2010) and in the presence of human serum (Jacobs et al., 2012), underscoring the ability of pathogens to quickly adapt to antibiotic challenge and exhibit an MDR phenotype. To tackle MDR *A. baumannii* isolates, either new antimicrobials are needed or a new strategy must be undertaken to restore sensitivity to conventional antimicrobials. The former option has limited feasibility as new antimicrobials, relative to drugs for metabolic disorders, are economically non-profitable, and in addition, the pathogen has the propensity to gain resistance even to new drugs due to evolutionary selection pressures, which further hinders the strategy. Hence, different approaches to resensitize existing conventional antimicrobials by using resistance modulators like efflux pump inhibitors, which cause an increased intracellular accumulation of antibiotics and reduce the propensity for gaining further resistance, appear promising.

Plant metabolites include a wide range of polyphenolic compounds such as flavonoids, coumarins, and tannins. They have been reported to exhibit efflux inhibitory potential against multiple microbial pathogens. For example, piperine and its derivatives are reported as efflux pump inhibitory against *Staphylococcus aureus* (Kumar et al., 2008), Similarly, capsaicin was shown to inhibit the NorA efflux pump in *S. aureus* (Kalia et al., 2012). Although many plant metabolites have been evaluated as efflux pump inhibitors predominantly against the NorA pump of *S. aureus* (Stavri et al., 2007), other reports have shown that curcumin, conessine, catharanthine, berberine, liquiritin, palmatine, caravilagenin C, theobromine, resveratrol, oleanolic acid, and osthol inhibit efflux pumps of Gram-negative pathogens like *E. coli* and *Pseudomonas aeruginosa* (Seukep et al., 2020). Ursolic acid has been shown to inhibit colistin efflux in clinical isolates of *K. pneumoniae* and *E. coli* (Sundaramoorthy et al., 2019). Similarly, curcumin, along with MarR inhibitor salicylate, restored colistin sensitivity in an XDR strain of *E. coli* (Sundaramoorthy et al., 2020). Tannic acid has been reported as inhibiting efflux in *A. baumannii* (Chusri et al., 2024). Hexane extract of *Acorus calamus L.* rhizome containing the metabolite asarone has been found to exhibit synergistic activity with ampicillin against *A. baumannii, Pseudomonas spp.,* and *Bacillus spp.* (Kongkham et al., 2024). Saleh et al. (2024) reported the downregulation of major RND efflux pumps when multiple clinical *A. baumannii* isolates were treated with cinnamon oil.

In the present study, 13 plant-derived polyphenols (quercetin, shikimic acid, piperine, myricetin, gallic acid, quinic acid, gallotannin, kaempferol, syringic acid, naringenin, caffeic acid, naringin, and picroside) were investigated for their potential to target the resistance nodulation division (RND) efflux pumps AdeA and AdeB of *A. baumannii*. An integrated *in silico* and *in vitro* strategy was employed, encompassing molecular docking and molecular dynamics simulations, followed by phenotypic efflux inhibition assays. The selected metabolites were further examined for their ability to modulate antibiotic activity *in vitro* and in an *in vivo* zebrafish infection model. Mechanistic studies were undertaken to elucidate the efflux inhibition-mediated potentiation of antibiotic efficacy.

## Materials and methods

### Bacterial strains and polyphenols

The reference strain of *Acinetobacter baumannii* (MTCC1425) was procured from the microbial type culture collection, MTCC, Chandigarh, India. Clinical isolates of *A. baumannii* (BC2267, U3145, E1406 and R232, R179) were obtained from Dr. Rangarajan Memorial Hospital, Sundaram Medical Foundation (SMF), Anna Nagar, Chennai India. These strains were grown on LB media at 37 °C. The media for bacterial culture and antibiotics were procured from Hi Media Labs, Mumbai, India. Fluorophores such as ethidium bromide (EtBr), 1-*N*-phenylethylamine (NPN), 3,3′-dipropylthiadicarbocyanine iodide [DiSC_3_(5)]), and 2′,7′-dichlorodihydrofluorescein diacetate (DCHF-DA) were purchased from Sigma Aldrich, Alfa Aesar, United States. Polyphenolic plant metabolites were either procured from Tokyo Chemical Industry Co., Ltd., Sisco Research Laboratories Pvt Ltd. (SRL), or HiMedia Laboratories, Mumbai, India. Various plant metabolites were evaluated in the study, including quercetin (>95% purity), shikimic acid (>97% purity), piperine, myricetin (>95% purity), gallic acid (98% purity), quinic acid (>98% purity), gallotannin, kaempferol (>95% purity), syringic acid (>95% purity), naringenin (>93% purity), caffeic acid (>98% purity), naringin (>95% purity), and picroside (>95% purity), respectively. Stocks of the polyphenols were freshly prepared in Milli-Q water and DMSO and stored at −20 °C for further use.

### Antimicrobial studies

Minimum inhibitory concentration (MIC) profiles of all the *A. baumannii* strains for various antibiotics and polyphenols were determined by the microbroth two-fold-dilution method (Andrews et al., 2001). In brief, overnight-grown cultures were adjusted to a 0.5 McFarland standard (40 µL of overnight culture diluted to 1 mL with sterile medium, corresponding to 10^5^–10^6^ CFU/mL). The standardized inoculum was then added to Mueller–Hinton broth (MHB) containing serially diluted antibiotics or test compounds and incubated at 37 °C. The MIC was determined by measuring optical density at 595 nm after 18–24 h of incubation. Similarly, the MICs of all polyphenols used in the study were determined against all *A. baumannii* isolates.

### Molecular docking

#### Protein and ligand preparation

The three-dimensional (3D) structures of the target proteins, including AdeA and AdeB, were retrieved from the RCSB Protein Data Bank (https://www.rcsb.org/) and UniProt (https://www.uniprot.org/). Protein structures were prepared by assigning appropriate bond orders and formal charges and by correcting missing hydrogen atoms and/or bonds to ensure reliability in docking simulations. All water molecules and non-essential heteroatoms were removed, except those critical for ligand–protein interactions. Ligand structures were obtained from chemical databases such as PubChem (https://pubchem.ncbi.nlm.nih.gov/) and downloaded in SDF format. Ionization states and relevant tautomeric forms were generated at physiological pH (7.4). The Epik tool was used to rank ligand conformations based on predicted pKa values, enabling identification of low-energy states that significantly influence docking accuracy. The prepared ligands were finally saved in “.mae” format for Glide docking (Gopikrishnan et al., 2023).

#### Binding site identification and grid generation

Protein binding pockets were identified using the CASTp (http://sts.bioe.uic.edu/castp/) and CASTpFold (https://cfold.bme.uic.edu/castpfold/) servers (Tian et al., 2018). The most suitable binding sites were selected based on the presence of druggable residues, as well as pocket surface area and volume. A docking grid box was generated using the grid generation tool, with interacting residues from the selected binding pocket manually specified to accurately define the grid. Molecular docking was subsequently carried out using the Schrödinger Protein Docking Tool (Gopikrishnan et al., 2023; Verliani et al., 2024).

#### Molecular docking

Molecular docking was performed using Glide (Schrödinger Suite), employing the extra precision (XP) method for the selection of optimal confirmations (Bhati et al., 2025), and the results were validated by Glide scores, a numerical estimate of binding affinity. Docking algorithms and settings included multiple runs, sampling various binding poses, and reporting Glide scores and docking scores (kcal/mol) for each ligand. For comparative analysis, docking runs were repeated for the all-tested polyphenols.

#### Analysis of docked complexes

The top-scoring complexes were visualized in Maestro and PyMOL (www.pymol.org), emphasizing hydrogen bonds, hydrophobic contacts, salt bridges, and π–π and anion–π interactions. Interacting residues, such as VAL14, ASN15, ILE16, GLN75, VAL78, GLN79, ILE82, LYS83, GLU86, LEU98, and LEU99, were documented and compared across ligands. A table summarizing Glide score, docking score, and interacting amino acids for each complex provided a way of choosing a suitable lead compound for further investigation. The 2D interactions were obtained from Maestro Schrödinger Software Suites, Maestro version 12.9 (Pauly et al., 2022), and the 3D interactions between ligands and proteins were visualized using PyMOL, version 3.1.6.1 (Yuan et al., 2016).

#### Molecular dynamics simulation

Molecular dynamics (MD) simulations were conducted using the GROMACS program with an AMBER-99SB force field (Wang et al., 2004). The simulations utilized the AdeB protein structure, with the ligand’s structure and electrostatic properties generated by ACPYPE. A dodecahedron box was fabricated, with the structural complex positioned at its center. The box was subsequently filled with TIP3P water molecules using the procedure described by Jorgensen et al. (1983). A minimum distance of 1 nm was established between the protein molecule and the boundary of the simulation box. GROMACS software application added counter-ions to achieve charge neutrality. A distance threshold of 14 Å was employed for non-covalent Van der Waals interactions. The LINCS method was employed to constrain covalent bonds involving hydrogen atoms (*LINCS: A Linear Constraint Solver for Molecular Simulations* - Hess - 1997 - Journal of Computational Chemistry - Wiley Online Library, 1998).

Energy was minimized using a step size of 0.001 nanoseconds. Afterward, a 100-picosecond simulation was performed in the isothermal–isovolumetric ensemble (NVT). Subsequently, a 10-nanosecond simulation was conducted using the isothermal–isobaric ensemble (NPT) to equilibrate the water system (Abraham et al., 2015). A molecular dynamics simulation was conducted using a non-polarizable tight-binding model. The simulation involved a 100-nanosecond production run. The simulation used a time step of 2 femtoseconds. The simulation employed a Parrinello–Rahman barostat and a modified Berendsen thermostat, maintaining a constant temperature of 300 K and pressure of 1 atm (Parrinello and Rahman, 1981). The root mean square deviation (RMSD) of the trajectory was calculated using GROMACS 2023.1 tools.

#### Synergy between polyphenols and antibiotics

MIC reversal assays for antibiotics in the presence and absence of polyphenols and standard efflux pump inhibitor PAβN (RND pump inhibitor) were performed for all *A. baumannii* clinical isolates to screen for the effective compound, which reversed the MIC of antibiotics in drug-resistant clinical isolates. In short, polyphenols/PAβN at sub-MIC concentrations were incubated with varying concentrations of erythromycin for 24 h at 37 °C. Following incubation, growth was measured at a wavelength of 595 nm using a UV-VIS spectrophotometer (Evolution 201, Thermo Fisher Scientific, Waltham, MA), and the fold reduction in erythromycin MIC was deemed to be the modulation factor (Lowrence et al., 2016).

To decipher whether the chosen polyphenol, gallotannin, exhibited synergistic, additive, or antagonistic effect, gallotannin and erythromycin were tested in combination at different concentrations by checkerboard assay against *A. baumannii* strains (Lowrence et al., 2016). Based on the data, a FIC (fractional inhibitory concentration) index value was calculated; if the FIC values were <0.5, the interaction was deemed synergistic; between 0.5 and 2.0, the interaction was additive; >2.0, the interaction was deemed antagonistic (Odds, 2003).

#### Real-time efflux study

To evaluate the efflux pump inhibitory potential of the chosen polyphenol, gallotannin, relative to standard inhibitor PAβN, real-time efflux studies were carried out in an XDR clinical isolate of *A. baumannii* BC2267 strain using ethidium bromide (EtBr) 1 μg/mL as a substrate. In brief, microbial cells were starved, then EtBr was added, followed by supplementation of the cells with 0.4% glucose to activate efflux. Fluorescence due to EtBr accumulation within the cells was measured at Ex = 530 nm and Em = 585 nm (Sundaramoorthy et al., 2018). The enhancement in EtBr fluorescent intensity within the cells due to treatment with gallotannin/standard EPI was deemed as the measure of efflux inhibition activity.

#### Time-dependent accumulation

XDR *A. baumannii* BC2267 strain was grown to 0.4 OD (mid-log phase). The cells were washed and resuspended in PBS and supplemented using 0.4% glucose. EtBr (1 μg/mL) was added to the cells and incubated for 30 min. Gallotannin/standard EPI (PAβN) (16 μg/mL) was added, and subsequently the accumulation of EtBr was immediately measured for the next 20 min with Ex 530 nm and Em 585 nm at 5 min intervals (Sundaramoorthy et al., 2020).

#### Time-kill assay

The ability of gallotannin to potentiate the bactericidal activity of erythromycin was evaluated against the XDR clinical isolate *A. baumannii* BC2267 using a time-kill assay (Grillon et al., 2016). Mid-logarithmic phase cultures were exposed to erythromycin (16 μg/mL) alone or in combination with gallotannin (16 or 32 μg/mL). Erythromycin (16 μg/mL) in combination with PAβN (16 μg/mL) was included as a positive control, while untreated cultures served as the growth control. At specified time points (0, 2, 4, 6, 8, and 24 h), samples were withdrawn, serially diluted, and plated on LB agar. Plates were incubated at 37 °C for 24 h, after which colony-forming units (CFU/mL) were enumerated. The bactericidal activity of the combination treatments was assessed relative to the positive control and individual treatments (Grillon et al., 2016).

#### Membrane permeability

1-*N*-phenylethylamine (NPN) uptake assay was used to assess the ability of gallotannin to permeabilize the outer membrane of XDR *A. baumannii* (Helander et al., 2000). As the LPS in the outer membrane (OM) of *A. baumannii* creates steric hindrance to hydrophobic molecules and restricts NPN entry; enhanced NPN fluorescence due to treatment with gallotannin/positive control colistin implies enhanced OM permeability. In brief, exponential-phase cells were collected, washed with 5 mM HEPES buffer containing 0.2% glucose at pH 7.5, and resuspended in an equal volume of the same buffer. NPN was added at a concentration of 0.5 mM, then gallotannin (16 μg/mL)/gallotannin + Erythromycin (16 μg/mL)/gallotannin + colistin was added. NPN fluorescence due to various treatments were measured (Ex = 350 and Em = 420 nm) using a multimode reader (Synergy H1M, Agilent, Santa Clara, CA). NPN in buffer and NPN in buffer along with cells were maintained as controls.

#### Membrane potential assay

Gallotannin and gallotannin–erythromycin combination were tested for the ability to perturb membrane potential, which was evaluated using the cationic membrane permeabilizing dye DiSC_3_(5). Accumulation of the dye in the lipid bilayer of intact cells results in the fluorescence being quenched. When the membrane becomes depolarized, the dye is released into the surrounding aqueous phase, resulting in enhanced fluorescence (Te Winkel et al., 2016). The fluorescence of DiSC_3_(5) in buffer was first measured at Ex = 610 ± 5 nm and Em = 660 ± 5 nm. Subsequently, exponential-phase cells were added, which subdued the fluorescence of DiSC_3_(5) due to the accumulation of dye within the lipid bilayer of intact cells. Protonophore CCCP was used as the positive control. Gallotannin (16 μg/mL)/CCCP treatments were given, and the resulting variation in fluorescence intensity due to various treatments were quantified using a multimode reader (Synergy H1M, Agilent, Santa Clara, CA).

#### ROS assay

Reactive oxygen species (ROS) generation in the XDR *A. baumannii* BC2267 strain following treatment with gallotannin or erythromycin alone, and gallotannin or PAβN in combination with erythromycin, was assessed using the fluorogenic probe 2′,7′-dichlorodihydrofluorescein diacetate (DCFH-DA). In brief, intracellular ROS production was quantified based on the oxidation of DCFH-DA to the fluorescent compound dichlorofluorescein (DCF). Fluorescence intensity was measured using a multimode reader (Synergy H1M, Agilent, Santa Clara, CA) at an excitation wavelength of 485 nm and an emission wavelength of 538 nm (Sundaramoorthy et al., 2019). Hydrogen peroxide was included as a positive control.

#### MTT assay

Gallotannin toxicity was evaluated in RAW 264.7 macrophages using an MTT assay. RAW macrophages were grown in DMEM with 10% FBS at 37 °C and 5% CO_2_. Once they attained 70% confluency, cells were trypsinized, resuspended in fresh media, and ∼10^6^ cells/well were seeded into 96-well plates. Following 24 h incubation, gallotannin at various concentrations (2–48 μg/mL) was added, and cells were incubated further for 24 h; subsequently, MTT (0.5 mg/mL) was added and incubated for 1 h. The formazan crystals formed by the metabolically active cells were dissolved with DMSO, and the absorbance of the extracted fraction was measured at 595 nm to assess cell viability (Peng et al., 2006).

#### Zebrafish infection

*In vivo* experiments were conducted in accordance with the guidelines of the Committee for the Purpose of Control and Supervision of Experiments on Animals (CPCSEA; Central Act 26 of 1982). All experimental protocols were reviewed and approved by the Institutional Animal Ethics Committee of SASTRA Deemed University, India (CPCSEA-510/SASTRA/IAEC/RPP). Zebrafish (*Danio rerio*) were used as the *in vivo* infection model. Intramuscular infection was established in zebrafish (n = 6 per group) using the XDR *A. baumannii* clinical isolate BC2267 at an optical density of 0.4, corresponding to approximately 1 × 10^8^ CFU/mL, following Neely et al. (2002) with minor modifications.

At 2 h post-infection, animals were treated with gallotannin or erythromycin alone or with a gallotannin–erythromycin combination, administered as a single intramuscular dose. At 24 h post-treatment, zebrafish were euthanized and decapitated, and the infected muscle tissue was aseptically excised, minced, serially diluted, and plated on LB agar. Following incubation for 24 h, bacterial burden was quantified by enumerating colony-forming units (CFUs). The reduction in bacterial bioburden achieved by gallotannin alone and in combination with erythromycin was determined based on CFU counts and represented graphically.

#### Dansyl-chloride-tagged erythromycin accumulation

To quantitatively assess the intracellular accumulation of erythromycin, the antibiotic was derivatized with dansyl chloride, enabling fluorescence-based detection. Dansyl-chloride-tagged erythromycin was used at a final concentration of 16 μg/mL. In brief, exponential-phase cells of *A. baumannii* BC2267 were harvested, washed with phosphate-buffered saline (PBS), and resuspended in an equal volume of the same buffer. Cell suspensions were then treated with dansyl-tagged erythromycin in combination with gallotannin, PAβN, or colistin for 30 min. Following treatment, cells were washed twice with PBS to remove extracellular antibiotic. The intracellular accumulation of dansyl-conjugated erythromycin under different treatment conditions was quantified by measuring fluorescence at an excitation wavelength of 335 nm and an emission wavelength of 515 nm using a multimode plate reader (Synergy H1M, Agilent, Santa Clara, CA).

#### Statistical analysis

All experiments were conducted in triplicate, and statistical analyses were performed using Student’s t-test or one-way ANOVA, as appropriate, with GraphPad Prism version 8.0 (GraphPad Software, La Jolla, CA, United States).

## Results

### Antimicrobial profiling of isolates

*A. baumannii* isolates were obtained from the blood/urine/ear of patients with infections from a tertiary healthcare setting. All 13 polyphenols exhibited a higher MIC of >256 μg/mL against *A. baumannii* isolates (Supplementary Table S1). The antimicrobial profiling of clinical isolates against 12 antibiotics belonging to different classes showed that the *A. baumannii* BC2267 strain has a high level of resistance to all 11 antimicrobials tested other than colistin and can be deemed an extremely drug-resistant (XDR) strain (Supplementary Table S2). In addition, the urinary isolate AB U3154 displayed resistance to multiple antimicrobials other than tetracycline and tobramycin. The MTCC AB1425 strain displayed resistance to four antimicrobials—erythromycin, streptomycin, amoxicillin, and meropenem—but exhibited either sensitivity or intermediary resistance to other tested antimicrobials (Supplementary Table S2). Thus, based on the antibiogram profile, AB BC2267 can be deemed an XDR strain as suggested by the International Expert Committee for Standard Definitions on Acquired Resistance (Magiorakos et al., 2012).

### Molecular docking studies

Molecular docking facilitates a comprehensive understanding of the intricate interactions between drug efflux proteins (AdeA and AdeB) and polyphenols. The Schrödinger Software Suite, Maestro version 12.9 was employed to identify and evaluate ligands with optimal Glide scores against key proteins involved in the AdeABC efflux pump mechanism—AdeA, a membrane fusion protein, and AdeB, a multidrug transporter (Gopikrishnan et al., 2023).

**FIGURE 1:**
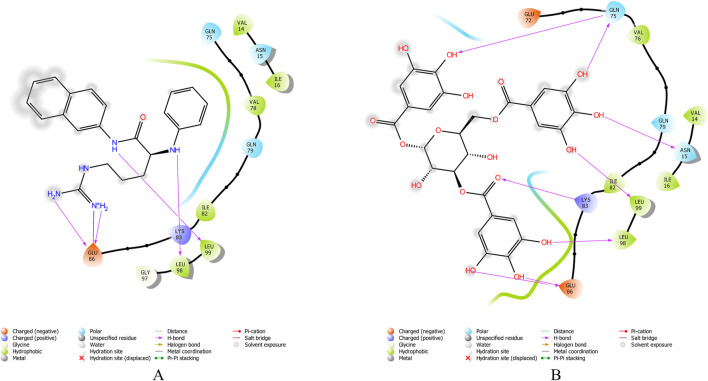
LigPlot shows that gallotannin relative to PAβN exhibits more favorable interactions with AdeB from *A. baumannii*: **(A)** AdeB interactions with Gal; **(B)** AdeB interaction with PAβN.

The 3D structure of proteins was downloaded from RCSB PDB and/or UniProt databases, and the structure prepared using the “Protein Prep” tool in Maestro 12.9. Binding pockets for the target protein were identified using the CASTp and CASTpFold servers. The optimal binding pocket was selected based on two criteria: (1) high surface area-to-volume ratio, and (2) location within the protein core. For the AdeA protein, the most suitable binding pocket exhibited a surface area of 21.897 Å^2^ and a volume of 5.809 Å^3^. The amino acid residues involved in pocket interactions are detailed in Supplementary Tables S3,S4. These data were used to generate the grid for proteins. Multiple conformations were generated from ligands using the LigPrep tool in Schrödinger. The best confirmations for ligands were screened using the Epik tool. The top ten ligand conformations were then selected for docking against the AdeA and AdeB proteins using the extra precision (XP) method. Figures 1A,B present 2D interactions of protein and ligands, and 3D interaction of the same were visualized using PyMOL (Supplementary Figure S1,S2). A series of polyphenols, along with the standard efflux pump inhibitor PAβN, were docked against the AdeA and AdeB proteins. Among the compounds tested, gallotannin demonstrated the highest binding affinity for both AdeA (Supplementary Table S3) and AdeB proteins (Supplementary Table S4), with Glide scores of −9.65 kcal/mol and −7.49 kcal/mol, respectively. These binding scores exceeded those of the standard inhibitor PAβN (Tables 1, 2) as well as all other tested polyphenols (Supplementary Tables S3, S4) with Glide scores of less than −5.0 kcal/mol. Notably, both PAβN and other polyphenols exhibited Glide scores of less than −5.0 kcal/mol. Based on the *in silico* observations, it is evident that gallotannin is a potential inhibitor of the AdeABC efflux pump in *A*. *baumannii*.

**TABLE 1:** Molecular docking of potent EPI gallotannin/standard EPI PAβN with AdeA pump of *A. baumannii*, highlighting interacting residues.

Two-dimensional structures of compound	Docking score	Glide score	Number of H-bonds	Interactive residues with interactive bonds
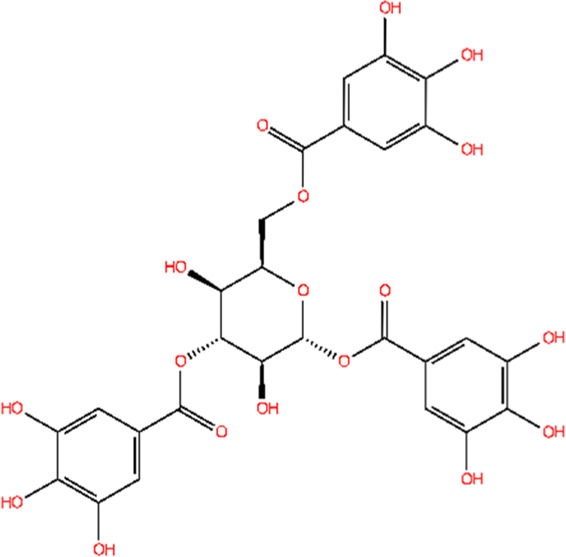 Gallotannin	−9.487	−9.658	8	GLU217, GLU338, ASN50, GLN335, TYR337, ASN46, SER48, and GLU49—conventional hydrogen bonds LEU221, ALA224, ARG220, SER214, GLN213, PRO52, LEU51, PHE47, and LEU230—other interactive residues
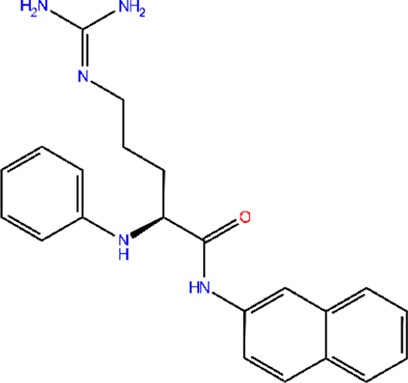 PAβN	−3.388	−3.388	2	GLU217—conventional hydrogen bond PHE47, SER48, GLU49, ASN50, LEU51, GLN213, ARG220, LEU221, ALA224, GLU229, LEU230, ILE296, and GLN335—other interactive residues

**TABLE 2:** Molecular docking of potent EPI gallotannin/standard EPI PAβN with AdeB pump of *A. baumannii*, highlighting interacting residues.

Two-dimensional structures of compound	Docking score	Glide score	Number of H-bonds	Interactive residues with interactive bonds
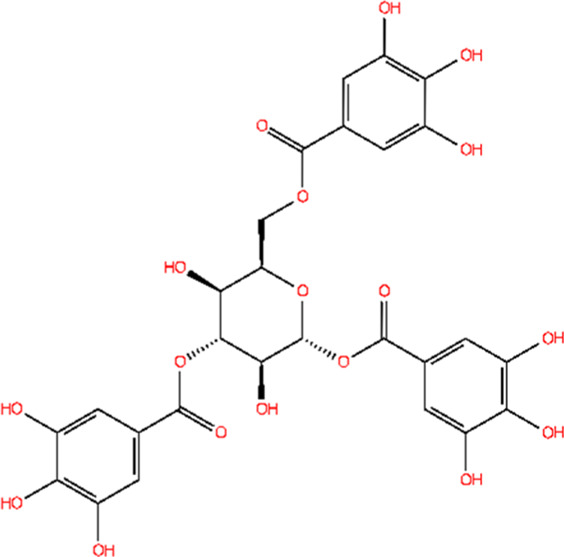 Gallotannin	−7.325	−7.496	8	LYS83, LEU99, GLU86, and GLN75—conventional hydrogen bonds; GLU72, VAL76, GLN79, ASN15, VAL14, ILE16, ILE82, and LEU98—other residues
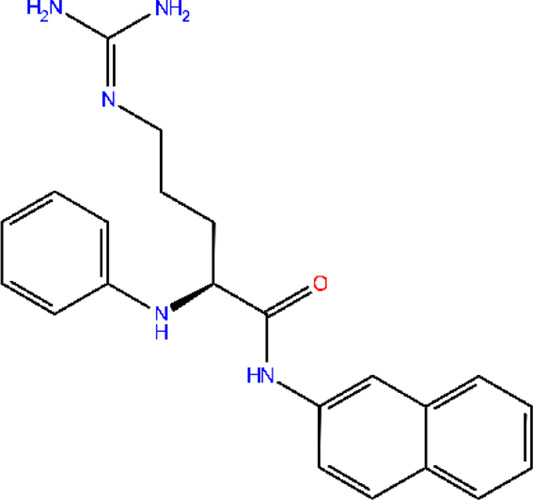 PAβN	−3.135	−3.135	4	LEU98, LEU99, and GLU86—conventional hydrogen bonds; GLN75, VAL14, ASN15, ILE16, VAL78, GLN79, ILE82, LYS83, and GLY 97—other residues

### Molecular dynamics simulation of interaction of gallotannin with AdeB

#### Root mean square deviation (RMSD)

**FIGURE 2:**
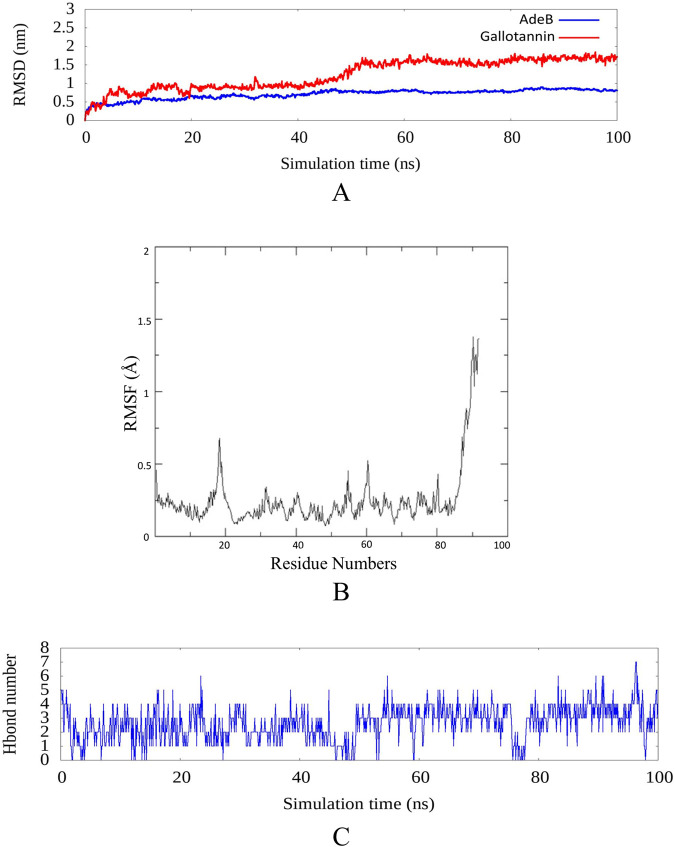
Molecular dynamics (MD) simulations of gallotannin with AdeB protein: **(A)** RMSD plot of the AdeB protein (blue) and the gallotannin–AdeB complex (red) over a 100 ns molecular dynamics simulation; upon gallotannin binding, a gradual increase in RMSD was observed during the initial phase of the simulation, suggesting conformational rearrangements within the binding pocket to accommodate the ligand. After approximately 50 ns, the RMSD reached a plateau, indicating that the system achieved a new equilibrium state. **(B)** RMSF plot of AdeB residues during the 100 ns MD simulation. The low RMSF values across most residues confirm that AdeB retains a stable conformation up to 80 residues. **(C)** Hydrogen bond profile of the gallotannin–AdeB complex. Gallotannin sustains stable binding with AdeB across the simulation, underscoring strong affinity and dynamic adaptability in the ligand–protein interface.

RMSD analysis highlights the overall structural stability of AdeB and its complex with gallotannin during the 100 ns MD simulation (Figure 2A). The unbound AdeB exhibited minimal fluctuations, maintaining a consistent backbone conformation which confirms the intrinsic stability of the protein. Upon gallotannin binding, a gradual increase in RMSD was observed during the initial phase of the simulation, suggesting conformational rearrangements within the binding pocket to accommodate the ligand. After approximately 50 ns, the RMSD reached a plateau, indicating that the system achieved a new equilibrium state. These results suggest that gallotannin binding induces moderate structural flexibility in AdeB without compromising its overall stability, supporting a stable and specific protein–ligand interaction (Baruah et al., 2022).

#### Root mean square fluctuation (RMSF)

The RMSF profile reveals that the majority of AdeB residues maintained low atomic fluctuations throughout the simulation, suggesting a stable and rigid core structure (Figure 2B). Localized peaks around residues 15–20 and 60–65 correspond to loop regions or surface-exposed segments that are inherently more flexible. Notably, a pronounced increase in RMSF is observed near the C-terminal region (residues 80–100), indicating significant flexibility in this segment. Such terminal mobility is common and is often associated with regions not directly involved in maintaining structural integrity or ligand binding. Overall, the low RMSF values across most residues confirm that AdeB retains a stable conformation up to 80 residue numbers. The observed fluctuations are confined to the peripheral C-terminal regions (Halder et al., 2023; Nyijime et al., 2025).

#### H-bond profile

Gallotannin exhibited persistent hydrogen bonding with AdeB throughout the molecular dynamics simulation, particularly within the 50–90 ns interval, where it consistently maintained at least four hydrogen bonds, indicative of steady interaction stability (Figure 2C). Notably, the highest number of hydrogen bonds observed was seven, occurring around the 96th nanosecond, while intermittent fluctuations in hydrogen bond count reflect inherent ligand–protein flexibility. Overall, these results demonstrate that gallotannin sustained stable binding with AdeB across the simulation, underscoring strong affinity and dynamic adaptability in the ligand–protein interface (Halder et al., 2023).

#### MIC reversal studies

The ability of 13 polyphenols (quercetin, shikimic acid, piperine, myricetin, gallic acid, quinic acid, gallotannin, kaempferol, syringic acid, naringenin, caffeic acid, naringin, and picroside) to reverse/reduce the MIC of erythromycin was evaluated against a reference strain of *A. baumannii* (MTCC1425) and an XDR clinical isolate (BC2267). Among the evaluated polyphenols, only gallotannin (1,2,6-tri-O-galloyl-bD-glucopyranose) caused a 64-fold MIC reversal of erythromycin against both isolates (Supplementary Table S5); hence, it was chosen for further studies. Subsequently, gallotannin was tested to reverse the MIC of antibiotics belonging to three different classes (cell wall inhibitor—meropenem, replication inhibitor—ciprofloxacin, and protein synthesis inhibitor—erythromycin) against five different clinical strains of *A. baumannii* and one reference strain, *A. baumannii* (MTCC1425). Gallotannin successfully reduced the MIC of all three antimicrobials against all the isolates of *A. baumannii* ∼64-fold (Table 3). In addition, we tested the ability of PAβN (standard efflux pump inhibitor) to reverse the MIC of these antibiotics against all six strains. Our observations showed that PAβN exerted significant reversal erythromycin MIC in the reference strain *A. baumannii* (MTCC1425) and two clinical isolates (BC2267& E1406), but for the other two antibiotics—meropenem and ciprofloxacin—PAβN failed to significantly (>2-fold) reverse the MIC of tested antibiotics against clinical isolates (Supplementary Table S6). As *A. baumannii* exhibits other mechanisms of resistance, such as OXA type Class D β-lactamase-mediated resistance for meropenem and gyrA/parC target site mutation mediated resistance for ciprofloxacin, efflux inhibition by PAβN (standard inhibitor) is unable to restore drug sensitivity in these isolates. It is noteworthy that gallotannin caused >64-fold MIC reversal for both meropenem and ciprofloxacin against five different clinical isolates (Table 3), implying that gallotannin apart from its efflux inhibitory potential also exhibits meropenem/ciprofloxacin resistance modulatory potential, which remains to be explored in further studies. Especially with ciprofloxacin, gallotannin fully restored the sensitivity of the fluoroquinolone in the XDR strain BC2267, MDR strains U3154 and 1,406, and the reference strain AB1425. Thus, by its synergistic interaction, Gallotannin succeeded in increasing the intracellular concentration of different antibiotics in five clinical isolates of *A. baumannii,* highlighting its strong efflux inhibitory potential. Erythromycin was chosen for further study since it is a less permeable macrolide antibiotic and previous studies have shown that erythromycin also exhibits biofilm inhibitory potential (Dong et al., 2024).

**TABLE 3:** Gallotannin reverses the MIC of three different antimicrobials in diverse *A. baumannii* strains: reference strain MTCC1415, clinical isolates: U3154, BC2267, 232, E1406, 179, ciprofloxacin (Cip), erythromycin (Ery), and meropenem (Mero).

Strain	MIC of erythromycin (μg/mL)	MIC of Gal + Ery (μg/mL)	MIC of ciprofloxacin (μg/mL)	MIC of Gal + Cip (μg/mL)	MIC of meropenem (μg/mL)	MIC of Gal + Mero (μg/mL)
MTCC1425	16	0.25	8	0.125	16	0.25
U3154	>256	4	128	2	>256	4
BC2267	>256	4	128	2	>256	4
232	>256	4	256	4	>256	4
E1406	>256	4	128	2	>256	4
179	>256	4	256	4	>256	4

#### Synergy testing

Among the clinical isolates of *A. baumannii* tested, since BC2267 was the sole XDR strain that displayed high levels of resistance to all the antimicrobials tested, further studies were carried out using it. Synergistic interaction between polyphenols and erythromycin was evaluated against the XDR BC2267 strain and among the polyphenols evaluated, and only gallotannin displayed synergy with the BC2267 strain with an FIC index of 0.125.

#### Real-time efflux (RTE) studies

To evaluate whether the MIC reversal caused by gallotannin is due to efflux inhibition, real-time efflux (RTE) and time-dependent accumulation (TDA) studies were performed. EtBr is a common substrate for most efflux transporters, and a compound inhibiting efflux will result in the enhanced accumulation of EtBr within the bacterial cells, which can be quantified using RTE/TDA. A real-time efflux assay was performed for the XDR BC2267 strain. Cells were initially starved by incubation in the absence of glucose. Subsequently, these cells were subjected to gallotannin/PaβN/Verapamil/PBS(Control) treatment for 1 h. Following this, the cells were supplemented with glucose, and fluorescence due to EtBr was measured for 20 min. RTE results revealed that gallotannin caused the enhanced inhibition of EtBr efflux relative to the positive controls PAβN (Standard RND pump inhibitor) (Nikaido and Pagès, 2012) and verapamil, a clinically approved calcium channel blocker used in the treatment of hypertension, which is widely employed as a standard inhibitor of MATE-family efflux pumps (Radchenko et al., 2015) (Supplementary Figure S4).

#### Time-dependent accumulation

**FIGURE 3:**
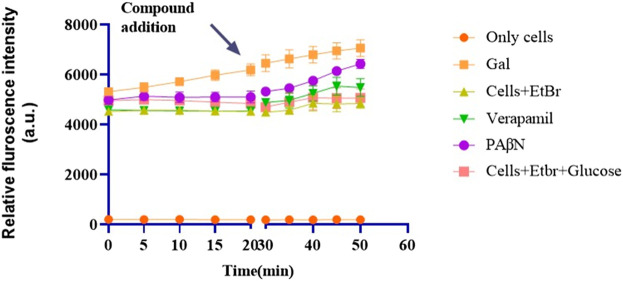
Gallotannin inhibits “efflux” and causes time-dependent accumulation of EtBr in *A. baumannii*. Mid-log cells of XDR *A. baumannii* BC2267 were incubated with EtBr for 20 min, and fluorescence followed for 20 min (Ex 530 nm and Em 585 nm). Post the addition of the compounds, fluorescence was measured for a further 20 min at 5 min intervals. Verapamil and PAβN were maintained as a positive control.

TDA focuses on net intracellular accumulation of EtBr within the bacterial cells. Cells supplemented with glucose tend to pump EtBr out through its efflux transporters. Efflux inhibition by gallotannin/standard inhibitors (PAβN and verapamil) leads to increased EtBr accumulation over time. The results (Figure 3) show that gallotannin causes a slight but enhanced accumulation of EtBr within XDR *A. baumannii* cells relative to positive control PAβN, which further corroborates the observation that gallotannin reverses the MIC of three different antimicrobials against five different clinical isolates of *A. baumannii* (Table 3).

#### Time-kill assay

**FIGURE 4:**
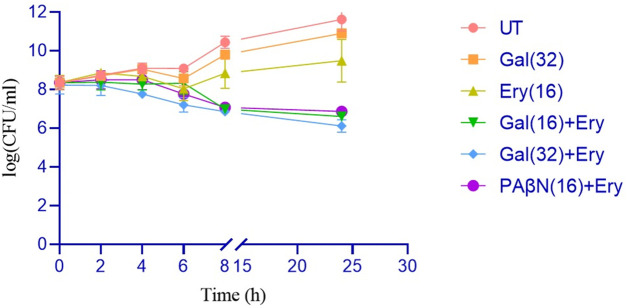
Gallotannin potentiates the bactericidal effects of erythromycin in the XDR isolate of *A. baumannii* BC2267. Mid-log phase cultures were treated with gallotannin (32 μg/mL) or erythromycin (16 μg/mL) alone, or with combinations of gallotannin (16 or 32 μg/mL) with erythromycin (16 μg/mL). A PAβN–erythromycin combination was included as a positive efflux inhibition control. Samples were collected at 0, 2, 4, 6, 8, and 24 h, serially diluted, and plated on LB agar for colony enumeration after 24 h of incubation. Viable counts were plotted over time. Statistical significance between treatment groups was assessed by two-way ANOVA with *P* < 0.001.

A time-kill assay was performed to evaluate the ability of gallotannin to potentiate the bactericidal effect of erythromycin. The concentrations of gallotannin and erythromycin employed for the time-kill assay were determined based on a checkerboard assay, which showed that the minimum effective concentration of gallotannin and erythromycin was 16 μg/mL. For the time-kill assay, mid-log cells of XDR *A. baumannii* BC2267 corresponding to 0.4 OD were subjected to the following treatments: gallotannin alone, erythromycin alone, gallotannin (16 μg/mL) + erythromycin (16 μg/mL), and gallotannin (32 μg/mL) + erythromycin (16 μg/mL); PAβN (16 μg/mL) + erythromycin (16 μg/mL) served as the positive control and the untreated culture served as the growth control. The plate counts at different time intervals revealed that treatment with gallotannin/erythromycin individually resulted in a trend similar to the untreated control, with a 2–3 log increase in cell counts by 24 h (Figure 4). Relative to treatment with erythromycin alone, a combination of gallotannin (16 μg/mL) with erythromycin resulted in ∼2 log decline by 24 h, which was similar to the reduction in cell counts induced by PAβN + erythromycin. However, a combination of 32 μg/mL of gallotannin with erythromycin (16 μg/mL) resulted in a significant 3 log decline, implying that gallotannin in combination with erythromycin enhanced the antimicrobial potential of erythromycin and effectively curtailed the growth of the XDR *A. baumannii* BC2267 strain.

As CRAB is a pressing issue with respect to nosocomial infections, we evaluated the ability of gallotannin to potentiate the bactericidal effect of meropenem (Carbapenem) in XDR *A. baumannii* strain using a time-kill assay (Supplementary Figure S5). Interestingly, meropenem treatment alone, like erythromycin, prevented the growth of *A. baumannii* but was unable to significantly reduce the founder population, whereas a gallotannin–meropenem combination caused a significant 2 log decline in cell counts, indicating that gallotannin by virtue of its efflux inhibitory potential causes increased the accumulation of meropenem, resulting in an enhanced bactericidal effect. These observations imply that a similar transporter could be involved in extruding both meropenem and erythromycin, the nature of which remains to be explored in future studies.

#### MTT assay to assess the toxicity of gallotannin

Gallotannin at various concentrations (2–48 μg/mL) was tested for its toxicity in raw macrophage cell lines. Approximately 10^6^ cells were exposed to different concentrations of gallotannin in triplicate, and after 24 h of incubation, cell viability due to treatment was assessed by MTT assay. The results (Supplementary Figure S6) revealed that gallotannin did not significantly impact the viability of macrophages; 80%–90% viability was retained at the different concentrations tested (2–32 μg/mL), and at 48 μg/mL, 70% viability was maintained. This indicates that gallotannin is relatively non-toxic to cultured raw macrophages, and at the concentration (16 μg/mL) employed throughout the present study, cells maintained ∼85–90% viability.

#### Zebrafish infection study

**FIGURE 5:**
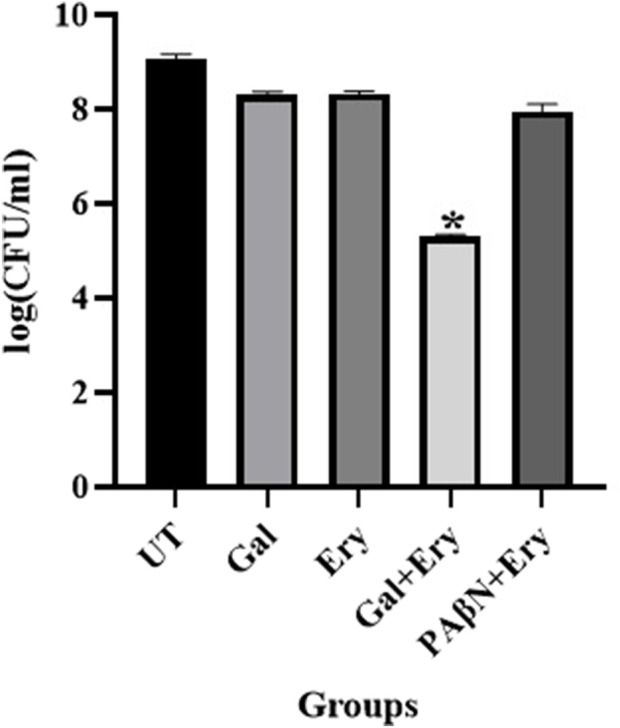
Gallotannin synergistically potentiates the bactericidal effect of erythromycin against XDR *A. baumannii* in infected zebrafish. Zebrafish were intramuscularly infected with 10^8^ CFU of *A. baumannii* BC2267; 2 h post-infection, the fish (n = 6 per group) were treated with gallotannin (Gal) or erythromycin (Ery) alone, or with combinations of Gal + Ery or PAβN + Ery. At 24 h post-treatment, the fish were euthanized, and the infected muscle tissue was dissected, homogenized, serially diluted, and plated on LB agar. Colony counts were determined after 24 h of incubation and expressed as log CFU/mL. Error bars represent the standard error of the mean (SEM) from six independent biological replicates. Statistical significance between treatment groups was assessed using Student’s t-test with *P* < 0.05.

To evaluate the ability of gallotannin to potentiate the antimicrobial effect of erythromycin against the XDR *A. baumannii* BC2267 strain, an *in vivo* zebrafish infection study was undertaken. Adult fish (n = 6) were independently infected with 0.4 OD of *A. baumannii* cells, corresponding to ∼1 × 10^8^ CFU/mL 2 h post-infection, and were treated with erythromycin alone, gallotannin alone, or a combination of erythromycin and gallotannin; a PAβN and erythromycin combination was used as positive control. Fish were euthanized 24–48 h post-infection, and muscle tissue was dissected, macerated, serial diluted, plated on LA plates, and incubated for 24 h at 37 ^°^C. Plate counts (Figure 5) revealed that the untreated control displayed a colony count of ∼9 log CFU. The gallotannin-treated group displayed ∼8 log CFU. Erythromycin treatment also resulted in colony counts comparable to gallotannin treatment. The positive control PAβN + erythromycin combination resulted in a minimal decline in cell counts ∼ 1 log decline in CFU relative to the untreated control. Among all treatments, gallotannin + erythromycin caused a significant 4 log decline in cell counts relative to the untreated control (Figure 5), underscoring the ability of gallotannin to potentiate the bactericidal effect of erythromycin *in vivo*. Despite the effectiveness of PAβN + erythromycin treatment in the time-kill assay, PAβN failed to potentiate the bactericidal effect of erythromycin in the zebrafish infection model for reasons unknown.

To gain mechanistic insights into the mode of action of gallotannin in synergizing the antibacterial potential of erythromycin, various assays like membrane permeability, intracellular accumulation using fluorescently tagged erythromycin, membrane potential, and ROS generation were undertaken.

#### Membrane permeability

**FIGURE 6:**
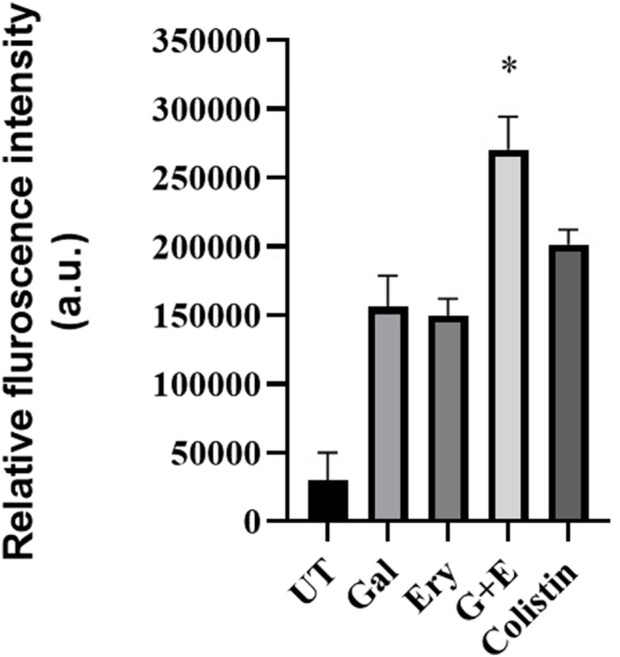
Gallotannin enhances the membrane permeability of XDR *A. baumannii*. Mid-log cells of *A. baumannii* BC2267 were exposed to N-phenyl 1-naphthylamine (NPN) and treated with either galloctannin (Gal), erythromycin (Ery) or their combination (Gal + Ery); colistin was used as positive control. The fluorescence intensity due to NPN (Ex = 350 nm and Em = 420 nm) was measured. The statistical significance between treatments was determined using Student’s t-test with P < 0.05.

Membrane permeability was determined using the fluorophore NPN. The results of the membrane permeability assay (Figure 6) revealed that untreated cells displayed baseline membrane permeability. Treatment with gallotannin caused a 6-fold increase in membrane permeability, similar to that caused by erythromycin. Positive control colistin induced an 8-fold increase in membrane permeability relative to untreated cells. However, treatment with a combination of gallotannin + erythromycin resulted in ∼10-fold increase in membrane permeability, indicating that gallotannin + erythromycin effectively permeabilizes the inner membrane of XDR *A. baumannii,* which also explains why gallotannin is effective in potentiating the bactericidal effect of erythromycin.

#### Accumulation of tagged erythromycin

**FIGURE 7:**
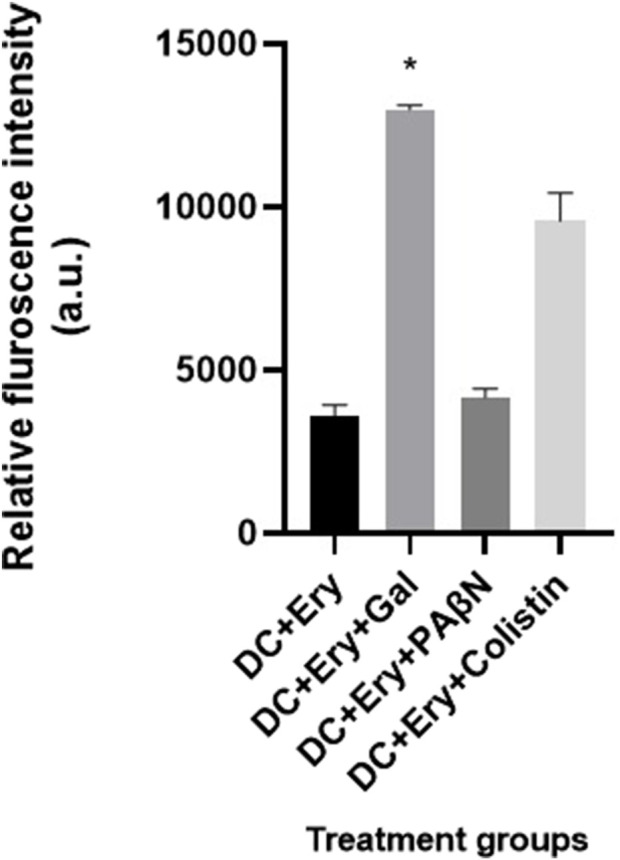
Gallotannin potentiates the accumulation of erythromycin in the XDR isolate of *A. baumannii* BC2267. Dansyl-chloride-tagged erythromycin was incubated with either gallotannin/PAβN/colistin for 30 min. Post incubation cells were washed and residual fluorescence due to dansyl-tagged erythromycin (Ex = 335 nm and Em = 515 nm) was quantified. The statistical significance between treatments were determined using Student’s t-test with P < 0.05.

In order to determine whether gallotannin causes the accumulation of erythromycin within the bacterial cells, erythromycin was coupled with dansyl chloride, incubated with *A. baumannii* cells, and co-treated with either gallotannin/PAβN/colistin for 30 min and followed by washing. The accumulation of dansyl-tagged erythromycin within the cells was quantified using a spectrofluorimeter. Cells treated with only dansyl-tagged erythromycin were maintained as a control. The results revealed that positive control colistin, which permeabilizes the membrane to facilitate erythromycin entry, caused ∼2-fold increase in fluorescence relative to the untreated control (Figure 7), whereas gallotannin caused a significant ∼3-fold increase in fluorescence due to the accumulation of tagged erythromycin within the cells (Figure 7). Interestingly, PAβN caused only a slight increase in tagged erythromycin accumulation relative to the untreated cells, which is not statistically significant. Thus, our observations indicate that gallotannin is actually effective in causing a significant accumulation of erythromycin within the cells, which could account for enhanced bactericidal effect of gallotannin-erythromycin combination.

#### ROS assay

**FIGURE 8:**
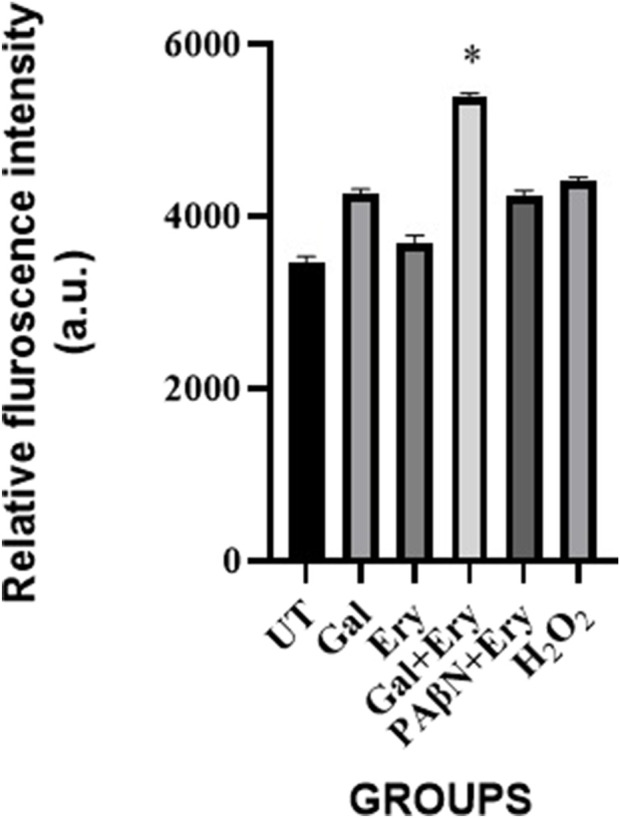
Gallotannin enhances erythromycin induced ROS generation in XDR *A. baumannii*. Mid-log phase cells of *A. baumannii* 2,267 strain were treated with galloctannin (Gal)/erythromycin (Ery) or gallotannin + erythromycin, PAβN + erythromycin. H_2_O_2_ was maintained as a positive control. ROS-mediated oxidation of DCHF-DA to DCF (Ex = 485 and Em = 530 nm) was measured. Statistical significance between treatments was determined using Student’s t-test with P < 0.05.

Many compounds exert an antibacterial effect by inducing ROS. Earlier studies have shown that, at least under aerobic conditions, conventional antibiotics induce ROS (Van Acker and Coenye, 2017). We were interested in exploring whether gallotannin exerts additional functional roles such as generating ROS apart from inhibiting drug efflux in *A. baumannii*. Our observations based on the ROS assay reveal that gallotannin caused ∼1.25-fold increase in ROS relative to untreated cells. As erythromycin is impermeable in Gram negatives, it did not alter ROS levels relative to untreated cells. When a combination of erythromycin and gallotannin was employed, a significant 1.5–1.8-fold increase in ROS was observed, which was greater than the ROS generation observed in cells treated with hydrogen peroxide (Figure 8). The observed enhancement in ROS could be attributed to two features of gallotannin: i) enhanced permeability allowing better access of erythromycin within bacterial cells and ii) efflux inhibition, which prevents the extrusion of accumulated erythromycin. Both these features generated sufficient ROS that could possibly attribute for the enhanced bactericidal effect of the gallotannin + erythromycin combination (Figures 4, 5). Positive control PAβN + erythromycin induced ROS comparable to H_2_O_2_ treatment but was unable to induce elevated ROS similar to gallotannin + erythromycin.

#### Membrane potential

A membrane potential assay was performed to evaluate whether gallotannin dissipated the proton motive force (PMF), which energizes proton-based efflux transporters. Protonophore CCCP was used as the positive control and DiSC_3_(5) as the probe. Membrane depolarization will result in removal of the dye from the lipid bilayer, leading to enhanced fluorescence. Treatment with gallotannin did not result in increased fluorescence, implying that, unlike protonophore CCCP, gallotannin did not cause membrane depolarization and probably does not inhibit efflux pumps by cutting off the energy source, like protonophore CCCP (Supplementary Figure S6).

## Discussion

We tested 13 different polyphenols for their ability to inhibit drug efflux in *Acinetobacter baumannii* by erythromycin MIC reversal assay. Erythromycin was chosen as the antibiotic of choice because the macrolide is relatively impermeable across Gram-negative cell walls (Mao et al., 1968), and microbial strains typically display resistance to it. In addition, erythromycin has been reported to display antibiofilm potential against *Porphyromonas gingivalis* (Maezono et al., 2011). A recent study has demonstrated that the microbial-derived cyclic lipopeptide brevicidine caused increased penetration of erythromycin and lowered the MIC of erythromycin in different *A. baumannii* isolates 32–128-fold (Zhong et al., 2023).

Among the polyphenols evaluated, gallotannin reversed the MIC of erythromycin > 64-fold against two different isolates of *A. baumannii* (Supplementary Table S3). Additionally, gallotannin also reversed the MIC of three different classes of antimicrobial—cell wall inhibitor (meropenem), protein synthesis inhibitor (erythromycin), and replication inhibitor (ciprofloxacin) —64-fold against five different MDR/XDR clinical isolates of *A. baumannii* (Table 3). Although PAβN reversed the MIC of erythromycin (Supplementary Table S4), it failed to significantly reverse the MIC of meropenem and ciprofloxacin in most of the clinical isolates. The ability of gallotannin to modulate the MIC of different classes of antimicrobials (Table 3) in clinical isolates of *A. baumannii*, along with pronounced EtBr accumulation as observed in time-dependent accumulation assays (Figure 3) and real-time efflux assays (Supplementary Figure S4), underscores its efflux inhibitory potential. Interestingly, gallotannin isolated from *Terminalia chebula* was previously reported in a short study to potentially inhibit efflux in MDR uropathogenic *E. coli* (Bag and Chattopadhyay, 2014). The authors had performed EtBr MIC reversal and MIC reversal of gentamycin and trimethoprim and observed a marginal two- to four-fold MIC reversal with gallotannin in MDR *E. coli* strain (Bag and Chattopadhyay, 2014). We used *A. baumannii,* which possesses a different array of efflux pumps than *E. coli*. Even among phylogenetically conserved RND pumps like AcrB from *E. coli* and AdeB from *A. baumannii,* which share 46% homology, the apo state of both pumps is quite different (Pos, 2024). The single-particle cryoEM structure of RND pumps shows that AdeB from *A. baumannii* exists in an improbable O–O–O apo (substrate-free) state (Su et al., 2019). This indicates that there is no binding site for substrates in this state and only one exit channel with an opening toward AdeA and AdeC. However, its homolog AcrB exists in L–L–L confirmation in the apo state (Murakami et al., 2002), indicating a readiness to bind drug by its open drug-binding access pocket. The apparent difference in confirmation could possibly be attributed to the differential permeability of the outer membrane (OM) of *E. coli* and *A. baumannii*. *E. coli* OM, being relatively more porous, permits accessibility to many drugs, and hence AcrB exists in ready L-conformation state to quickly pick up incoming drugs. In *A. baumannii*, due to the significantly reduced permeability of the OM, AdeB need not exist in L-conformation and instead exists in O-confirmation (Pos, 2024). Thus, comparison of the crystal structures of homologous pumps indicates that the two organisms could differ widely in their substrate specificity and extrusion mechanisms. In addition, relative to the earlier report, which showed a modest 2–4-fold MIC reversal in *E. coli* with gallotannin, we observed a drastic reduction in MIC of >32–64-fold with three different classes of antimicrobials against multiple clinical isolates of *A. baumannii* (Table 3). Molecular dynamics simulation studies showed that gallotannin exhibited persistent hydrogen bonding with AdeB throughout (Figure 2C). Gallotannin binding induced moderate structural flexibility in AdeB without compromising its overall stability (Figure 2A). These *in silico* observations reinforced the efflux inhibitory potential of gallotannin by stably interacting with the drug transporter AdeB.

Mechanistic explorations revealed that gallotannin enhanced erythromycin permeability (Figure 6). Zhong et al. (2023) showed that the microbial cyclic lipopeptide brevicidin enhanced erythromycin permeability in *A. baumannii* cells. In this study, gallotannin in combination with erythromycin caused a much higher enhancement in membrane permeability relative to treatment with the positive control, colistin (Figure 6). Gallotannin’s potential to induce enhanced permeability coupled with efflux inhibition (Supplementary Figure S4) is likely to result in the elevated intracellular accumulation of erythromycin, resulting in cell death. In agreement with this premise, our observations with dansyl-tagged erythromycin revealed that gallotannin caused a 3-fold enhanced accumulation of tagged erythromycin relative to the untreated control (Figure 7), which was better than the accumulation affected by the positive control, colistin. However, PAβN induced the slightly elevated accumulation of the tagged erythromycin relative to the untreated control (tagged erythromycin alone) (Figure 7). Despite the claim in the literature that erythromycin fails to penetrate cell walls of Gram-negative bacterium, our observations with tagged erythromycin (Figure 7) imply that a basal level of erythromycin accumulation occurs, which is slightly enhanced by PAβN treatment. This could account for the mild reduction in cell counts mediated by PAβN treatment. Phenylalanine–arginine β-naphthylamide (PAβN) inhibits the RND efflux system in Gram-negative bacteria by competitively binding to the substrate-recognition sites of the inner membrane transporter, thereby preventing antibiotic extrusion and enhancing intracellular drug accumulation (Nikaido and Pagès, 2012).

Most compounds exert their antibacterial effect via ROS. Although gallotannin treatment alone did not significantly elevate ROS, gallotannin in combination with erythromycin caused a drastic elevation in ROS, which was greater than ROS generated by hydrogen peroxide (Figure 8). These observations imply that gallotannin by virtue of its efflux inhibition causes enhanced intracellular accumulation of erythromycin, which in turn leads to elevated ROS levels. Kaur et al. (2018) had shown that a combination of polyphenolic plant metabolite curcumin with colistin significantly reduced persister cells of *A. baumannii* by significantly upregulating ROS, in addition to downregulating repair genes and inhibiting colistin efflux. Thus, by increasing erythromycin’s access to the cells and by inhibiting its extrusion, gallotannin significantly potentiates the bactericidal effect of erythromycin. In the time kill assay when gallotannin was applied at 16 μg/mL, the antibacterial effect of the gallotannin–erythromycin combination was comparable to that of the PAβN–erythromycin combination, resulting in an approximately 2-log reduction in viable cell counts relative to the initial (founder) population. Increasing the gallotannin concentration to 32 μg/mL further enhanced the combinatorial effect, leading to an approximately 3-log reduction in cell counts after 24 h (Figure 4). Gallotannin also caused a similar 3-log decline in CFU with meropenem in the time-kill assay (Supplementary Figure S5). These observations gain significance because of the high prevalence of CRAB in hospital-acquired ICU infections (41.7/1,000 patients with 95% CI 21.6–78.7) in Europe, the Eastern Mediterranean, and Africa, as reported in a metadata analysis study by Ayobami et al. (2019). In addition, treatment options against *A. baumannii* are severely limited, and controlled trials to guide therapeutic choices are scarce (Maragakis and Perl, 2008). Importantly, during the *in vivo* zebrafish infection studies, only the gallotannin + erythromycin combination caused a significant 4-log decline in cell counts (Figure 5). Surprisingly, the PAβN erythromycin combination, which was effective in the time-kill assay, failed to reduce bacterial bioburden in infected zebrafish (Figure 5), the reasons for this being unclear. The gallotannin–erythromycin combination was effective both *in vitro* and *in vivo*. Moreover, gallotannin displayed a lower cytotoxicity profile in the MTT assay (Supplementary Figure S6). Overall, our findings underscore the therapeutic potential of gallotannin as an adjuvant with erythromycin in tackling MDR *A. baumannii* strains.

Despite these promising findings, this study has the following limitations. The exact efflux transporter inhibited by gallotannin remains to be elucidated by qPCR and confirmed by pump knock-out and overexpression strains. Although we have shown that gallotannin works effectively against the XDR *A. baumannii* strain in time-kill and zebrafish infection studies, the potential of the combination to curtail the growth of all clinical isolates in *in vitro* and *in vivo* infection studies will add further value to these findings.

Given the renewed need for novel antimicrobial adjuvants to tackle MDR pathogens, our findings support the use of polyphenol gallotannin as a viable EPI against MDR *A. baumannii*. Its natural origin, *Terminalia chebula*, has an established usage and lack of toxicity by MTT assay (Supplementary Figure S6), and its ability to potentiate antimicrobial effect of erythromycin via enhanced permeability (Figure 6), synergistic ROS generation (Figure 8), and reduced extrusion of erythromycin by efflux inhibition (Supplementary Figure S4) makes gallotannin an attractive candidate for incorporation into combination therapy strategies, especially in face of increasing AMR strains and dwindling antibiotic discovery. Thus, the gallotannin–erythromycin combination has the potential to be evaluated in higher animal models to establish its therapeutic efficacy.

## Conclusion

Gallotannin was identified as an effective efflux pump inhibitor capable of restoring erythromycin activity against *Acinetobacter baumannii*. Efflux inhibition was consistently observed across five clinical isolates, and gallotannin exhibited strong synergistic interactions with multiple antibiotics, resulting in a 32–64-fold reduction in MICs. The resistance-modulating activity of gallotannin was primarily mediated through efflux pump inhibition, with additional contributions from increased membrane permeability, enhanced intracellular antibiotic accumulation, and the induction of reactive oxygen species. The gallotannin–erythromycin combination displayed superior bactericidal activity, achieving an approximately 3-log reduction in bacterial counts at 24 h in time-kill assays. *In vivo* validation using a zebrafish infection model further demonstrated a significant 4-log reduction in bacterial bioburden following combination therapy. Molecular docking and dynamics simulations revealed high binding affinity and stable interactions of gallotannin with AdeA and AdeB components of the AdeABC efflux system. Importantly, gallotannin did not significantly affect macrophage viability, underscoring its favorable safety profile. Overall, gallotannin represents a promising antibiotic adjuvant with considerable translational potential, meriting further evaluation in higher animal models.

## Data Availability

The original contributions presented in the study are included in the article/Supplementary Material; further inquiries can be directed to the corresponding authors.
