# Supplementary material for: Evaluation of the efflux inhibitory potential of gallotannin to restore drug sensitivity in XDR Acinetobacter baumannii in vitro, and a zebrafish infection model
Source: Front Pharmacol. 2026 Apr 8;17:1652777. doi: 10.3389/fphar.2026.1652777 (PMC13099881; doi:10.3389/fphar.2026.1652777)
Supplement: Supplementary file 1 [file Supplementaryfile1.docx]

**Supplementary Information**

**Table S1: MIC of plant Metabolites against Reference and Clinical isolate of *A. baumannii***

| **Polyphenols** | **AB 2267**  (XDR clinical isolate) | **AB 1415**  (Ref Isolate) |
| --- | --- | --- |
|  | MIC(μg/ml) | |
| Quercertin | >256 | >256 |
| Shikimic acid | >256 | >256 |
| Piperine | >256 | >256 |
| Myricetin | >256 | >256 |
| Gallic acid | >256 | >256 |
| Quinic acid | >256 | >256 |
| Gallotannin | >256 | >256 |
| Kaempferol | >256 | >256 |
| Syringic acid | >256 | >256 |
| Naringenin | >256 | >256 |
| Caffeic acid | >256 | >256 |
| Naringin | >256 | >256 |
| Picroside | >256 | >256 |

**Table S2: MIC Profile of different antimicrobials against reference and clinical isolates of *A. baumannii.* (R-Resistant, S-Sensitive).**

| **Antibiotics** | **AB U3154** | **AB R232** | **AB R179** | **AB E1406** | **AB BC2267** | **AB 1425** |
| --- | --- | --- | --- | --- | --- | --- |
|  | MIC(μg/ml) | | | | | |
| Erythromycin | >256(R) | >256(R) | >256(R) | >256(R) | >256(R) | 16(R) |
| Ciprofloxacin | 128(R) | 256(R) | 256(R) | 128(R) | 128(R) | 8(R) |
| Meropenem | >256(R) | >256(R) | >256(R) | >256(R) | >256(R) | 16(R) |
| Amoxicillin | >256(R) | >256(R) | >256(R) | >256(R) | >256(R) | >256(R) |
| Levofloxacin | 16(R) | 32(R) | 8(R) | 64(R) | >256(R) | <4(S) |
| Tetracycline | <4(S) | >256(R) | >256(R) | <4(S) | >256(R) | <4(S) |
| Tobramycin | <4(S) | <4(S) | >256(R) | >256(R) | 256(R) | <4(S) |
| Kanamycin | >256(R) | >256(R) | >256(R) | >256(R) | >256(R) | 8(R) |
| Amikacin | >256(R) | >256(R) | >256(R) | 128(R) | >256(R) | 4(S) |
| Gentamycin | >256(R) | >256(R) | >256(R) | >256(R) | >256(R) | 4(S) |
| Colistin | 32(R) | <4(S) | <4(S) | <4(S) | <4(S) | <4(S) |
| Streptomycin | 256(R) | >256(R) | >256(R) | >256(R) | >256(R) | 256(R) |

**Table S3: Molecular Docking of polyphenolics with AdeA RND pump of *A. baumannii***

| Two Dimensional Structures of Compound | Compound Name | PubChem ID | Docking Score | Glide Score | No. of  H-Bonds | Interactive Residues with Interactive Bonds |
| --- | --- | --- | --- | --- | --- | --- |
| 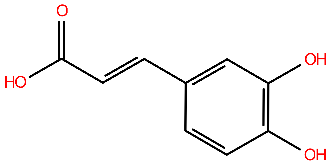 | Caffeic Acid | 689043 | -3.000 | -3.000 | 3 | GLN335, GLU49 – Conventional H-Bonds;  GLU229, LEU230, ILE296, ALA294, ARG293, PHE47,  GLU217, GLN213, ARG220, LEU221, ALA224 – Other Interacting Residues |
| 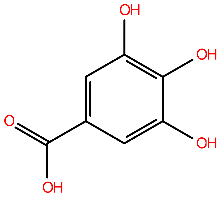 | Gallic Acid | 370 | -4.597 | -4.604 | 3 | GLN335, GLU49 – Conventional H-Bonds; LEU230, PHE47, GLU217, GLN213, ARG220, LEU221, ALA224 – Other Interacting Residues |
| 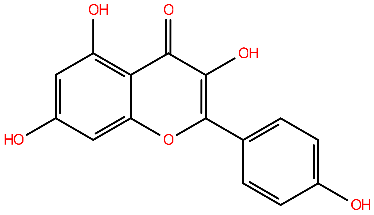 | Kaempferol | 5280863 | -3.570 | -3.602 | 3 | GLU49, GLU217, GLU229 – Conventional H-Bonds; LEU230, ALA294, PHE47, ALA224, LEU221, ARG220, GLN335 – Other Interacting Residues |
| 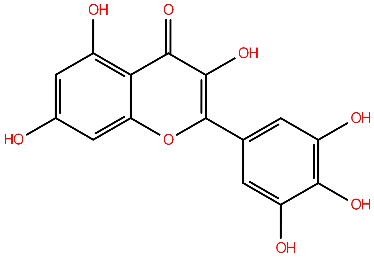 | Myricetin | 5281672 | -4.374 | -4.411 | 4 | GLU49, GLU217, GLU229 – Conventional H-Bonds; LEU230, PHE47, ALA224, LEU221, ARG220, GLN335 – Other Interacting Residues |
| 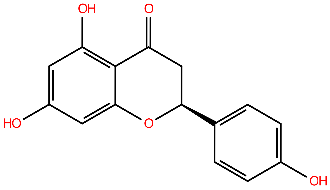 | Naringenin | 439246 | -3.324 | -3.342 | 2 | GLU49, GLU229 – Common H-Bonds; LEU230, PHE47, ARG293, ALA294, GLN335, ARG220, LEU221, ALA224 – Other Interacting Residues |
| 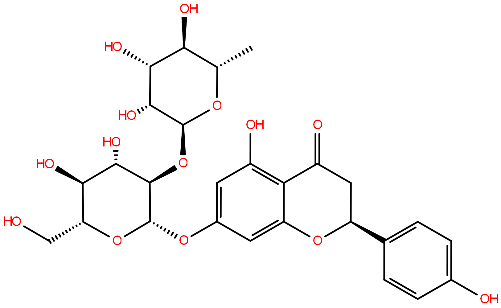 | Naringin | 442428 | -5.122 | -5.122 | 6 | TYR341, GLN336, GLY334, ILE333, GLU49 – Conventional H-Bonds; ASN344, GLN335, LEU230, PHE47, ARG293, ALA294, ALA224, LEU221, ARG220 – Other Interacting Residues |
| 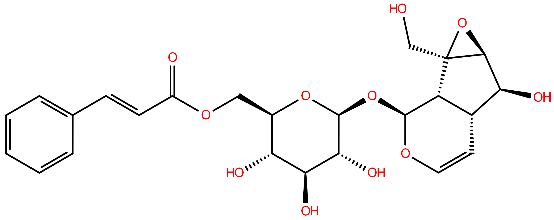 | Picroside | 6440892 | -4.158 | -4.158 | 4 | GLU49, GLU217, GLN335, GLN336 – Conventional H-Bonds; GLU338, TYR337, GLY334, ILE333, LEU230, GLU229, PHE47, SER48, GLN213, ARG220, LEU221, ALA224 – Other Interacting Residues |
| 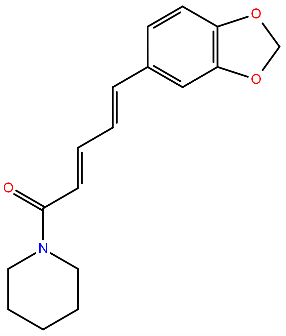 | Piperine | 638024 | -2.318 | -2.318 | 0 | GLU338, TYR337, GLN336, GLN335, ALA294, LEU230, PHE47, SER48, GLU49, GLU217, GLN213, ARG220, LEU221, ALA224 – Other Residues |
| 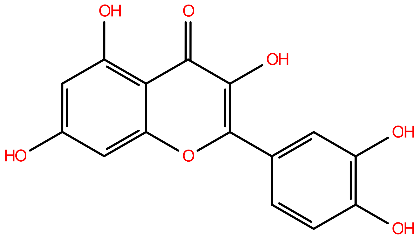 | Quercertin | 5280343 | -3.469 | -3.501 | 4 | GLU49, GLU338, GLN335 – Conventional H-Bonds; TYR337, GLN336, PHE47, SER48 – Other Interacting Residues |
| 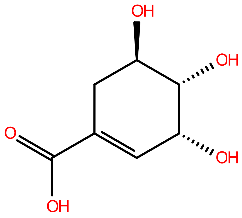 | Shikimic Acid | 8742 | -4.730 | -4.731 | 3 | GLN335, GLU49 – Conventional H-Bonds; ALA294, ARG293, PHE47, GLU217, GLN213, ARG220, LEU221, ALA224, LEU230 – Other Interacting Residues |
| 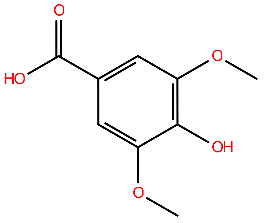 | Syringic Acid | 10742 | -2.950 | -2.950 | 1 | GLN335 – Conventional H-Bond; GLU229, LEU230, ILE296, ALA294, ARG293, PHE47, GLU49, GLU217, GLN213, ARG220, LEU221, ALA224 – Other Interacting Residues |

**Table S4. Molecular Docking of polyphenolics with AdeB RND pump of *A. baumannii***

| Two Dimensional Structures of Compound | Compound Name | PubChem ID | Docking Score | Glide Score | No. of  H-Bonds | Interactive Residues with Interactive Bonds |
| --- | --- | --- | --- | --- | --- | --- |
| 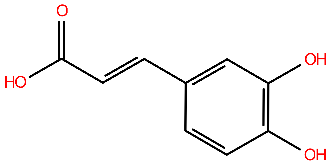 | Caffeic Acid | 689043 | -2.015 | -2.015 | 2 | ASN15, GLN79 – Conventional H-Bonds, GLU86, LYS83, ILE82, VAL78, VAL14, ILE16, LEU98, LEU99 – Other Interactive Residues |
| 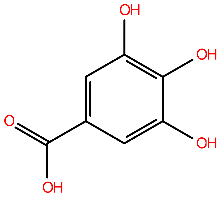 | Gallic Acid | 370 | -3.741 | -3.748 | 2 | LEU98 – Conventional H-Bonds, VAL14, ASN15, ILE16, GLN75, VAL78, GLN79, ILE82, LEU99 – Other Interactive Residues |
| 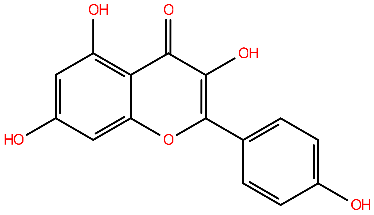 | Kaempferol | 5280863 | -2.804 | -2.836 | 2 | ASN15, GLU86 – Conventional H-Bonds; LYS83, ILE82, GLN79, VAL78, ILE16, VAL14, GLN75, LEU98, LEU99 – Other Interactive Residues |
| 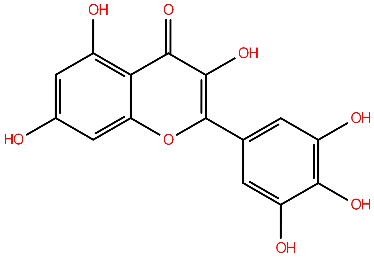 | Myricetin | 5281672 | -4.475 | -4.512 | 2 | ASN15, GLU86 – Conventional H-Bonds; VAL14, ILE16, GLN75, VAL78, GLN79, ILE82, LYS83, GLY97, LEU98, LEU99 – Other Interactive Residues |
| 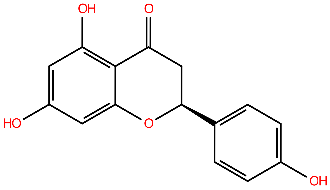 | Naringenin | 439246 | -2.589 | -2.607 | 2 | ASN15, LYS83 – Conventional H-Bonds; GLU86, ILE82, GLN79, VAL78, VAL14, ILE16, LEU98, LEU99 – Other Interactive Residues |
| 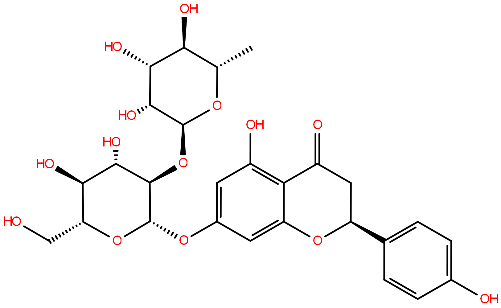 | Naringin | 442428 | -3.181 | -3.181 | 3 | ASN15, GLU86 – Conventional H-Bonds; LYS83, ILE82, GLN79, VAL78, GLN75, ILE16, VAL14, LEU98, LEU99 – Other Interactive Residues |
| 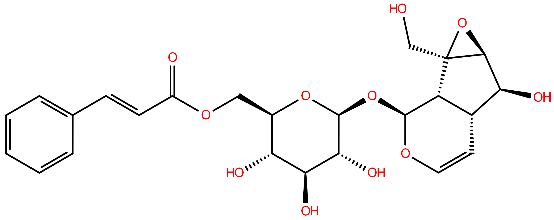 | Picroside | 6440892 | -3.261 | -3.261 | 5 | LEU99, GLN79, GLN75, GLU72 – Conventional H-Bonds; GLU86, LYS83, ILE82, VAL78, VAL76, ILE16, ASN15, VAL14, LEU98 – Other Interactive Residues |
| 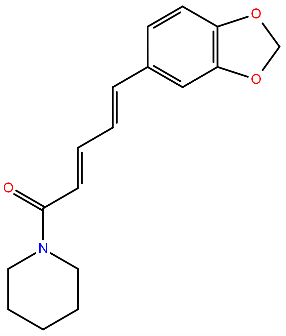 | Piperine | 638024 | -1.626 | -1.626 | 1 | LEU98 – Conventional Hydrogen Bond; GLU86, LYS83, ILE82, GLN79, VAL78, GLN75, ILE16, ASN15, VAL14, GLY97, LEU99 – Other Interactive Residues |
| 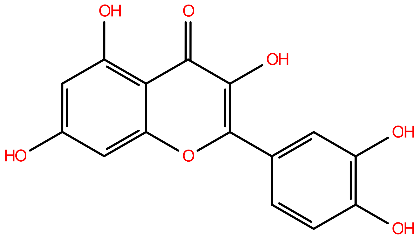 | Quercertin | 5280343 | -3.369 | -3.401 | 3 | ASN15, GLN79, GLU86 – Conventional H-Bonds; ILE16, VAL14, VAL78, ILE82, LYS83, GLY97, LEU98, LEU99, ARG94 – Other Interactive Residues |
| 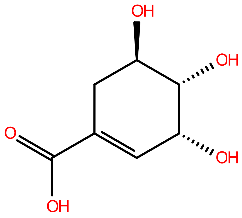 | Shikimic Acid | 8742 | -4.861 | -4.862 | 3 | LEU98, GLU46 – Conventional H-Bonds; LYS83, ILE82, GLN79 – Other Interactive Residues |
| 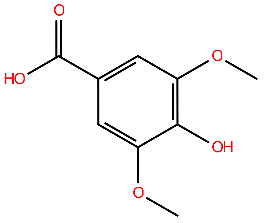 | Syringic Acid | 10742 | -2.670 | -2.670 | 2 | ASN15, GLN79 – Conventional H-Bonds; VAL14, LEU99, ILE16, VAL78, GLN75 – Other Interactive Residues |

| **Polyphenols** | **AB 2267** | **AB 1415** |
| --- | --- | --- |
|  | Modulation factor with erythromycin | |
| Quercertin | 1 | 1 |
| Shikimic acid | 1 | 1 |
| Piperine | 1 | 1 |
| Myricetin | 1 | 1 |
| Gallic acid | 1 | 1 |
| Quinic acid | 1 | 1 |
| Gallotannin | 64 | 64 |
| Kaempferol | 1 | 1 |

**Table S5:**  **Gallotannin exclusively reverses MIC of erythromycin in *A. baumanii* reference strain MTCC1415 and XDR clinical isolate of *A. baumannii* BC2267**

| **Polyphenols** | **AB 2267** | **AB 1415** |
| --- | --- | --- |
|  | Modulation factor with erythromycin | |
| Syringic acid | 1 | 1 |
| Naringenin | 1 | 1 |
| Caffeic acid | 1 | 1 |
| Naringin | 1 | 1 |
| Picroside | 1 | 1 |

**Table S6: Ability of PAβN to reverse MIC of 3 antimicrobials against reference strain MTCC1415 and Clinical isolates of *A. baumannii*.**

| **STRAINS** | **MIC Reversal of Erythromycin (**μg/ml**)** | **MIC Reversal of Meropenem**  **(**μg/ml**)** | **MIC Reversal of Ciprofloxacin (**μg/ml**)** |
| --- | --- | --- | --- |
| MTCC1425 | 64 | 64 | 64 |
| U3154 | 1 | 2 | 1 |
| BC2267 | 32 | 2 | 1 |
| 232 | 2 | 2 | 1 |
| E1406 | 4 | 1 | 2 |
| 179 | 1 | 1 | 1 |

**Figure S1: Three-dimensional interaction of A-Gallotannin, B-PaβN with AdeA RND pump of *A. baumannii.***


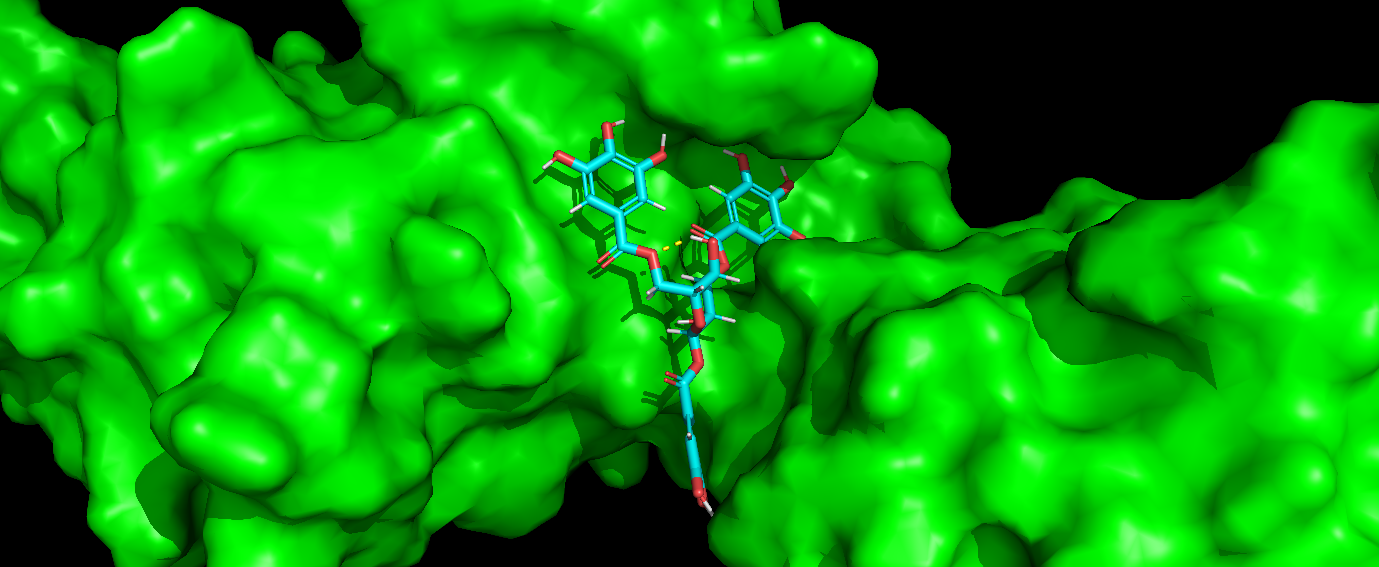


**(A)**


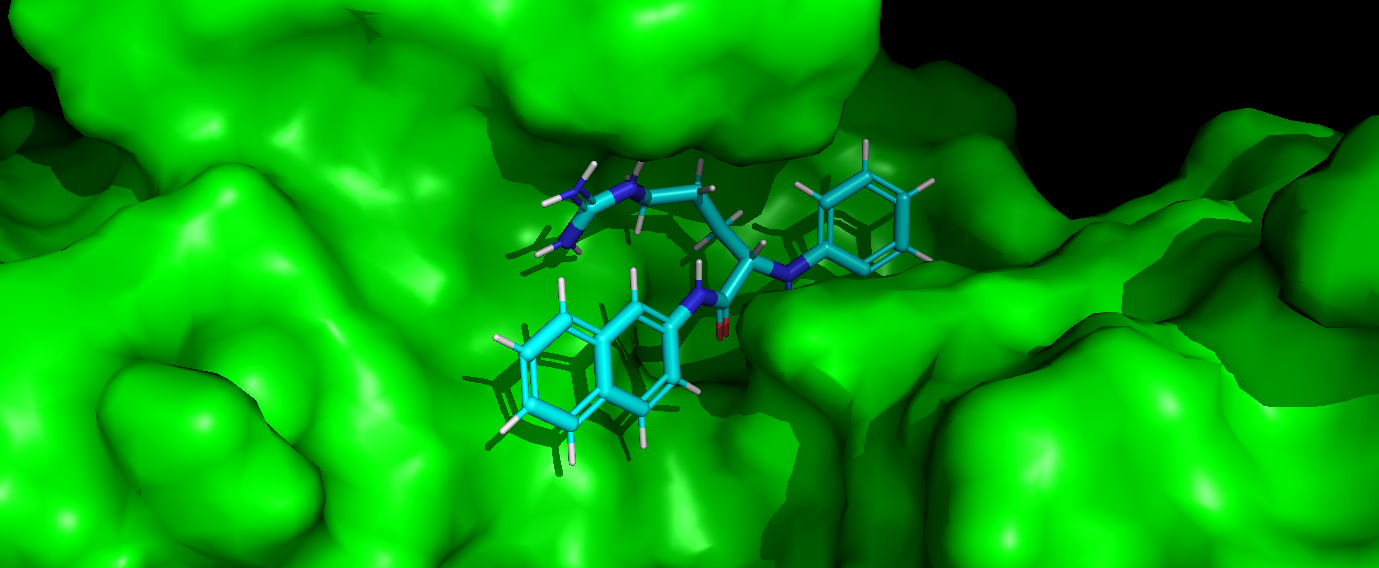


**(B)**

**Figure S2: Three-dimensional interaction of A-Gallotannin, B-PaβN with AdeB RND pump of *A. baumannii.***


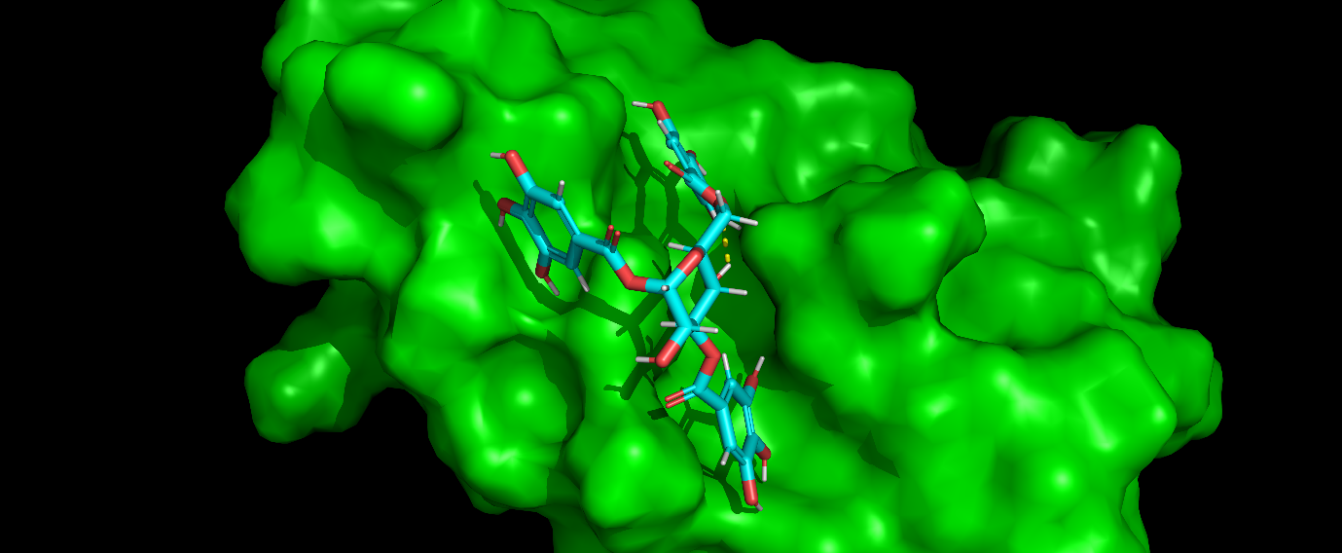


**(A)**


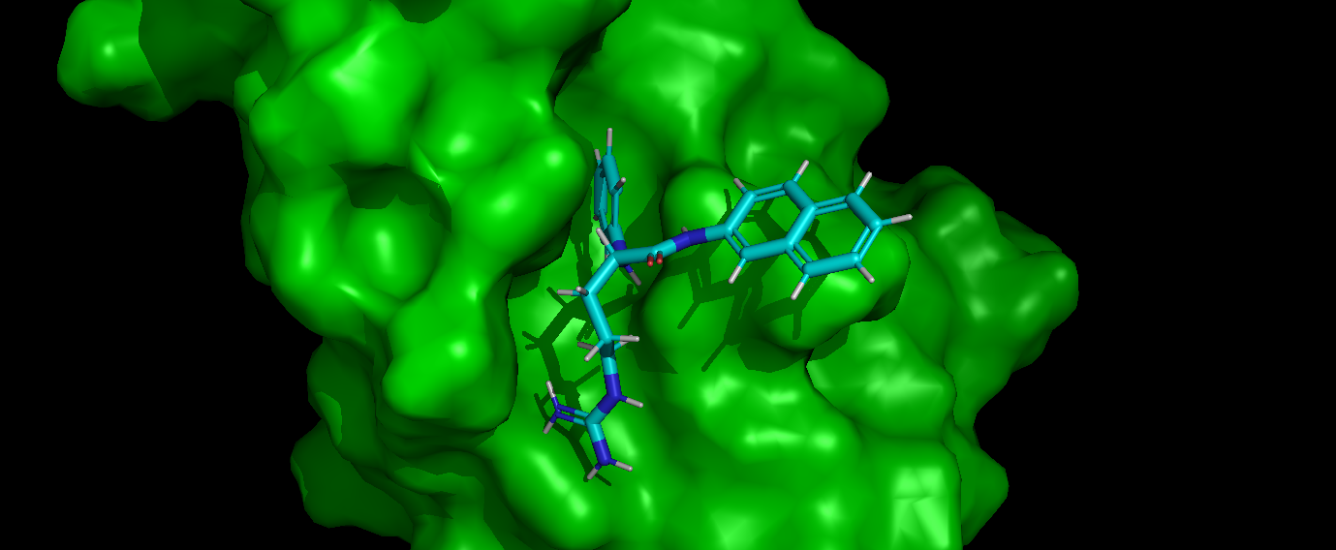


**(B)**


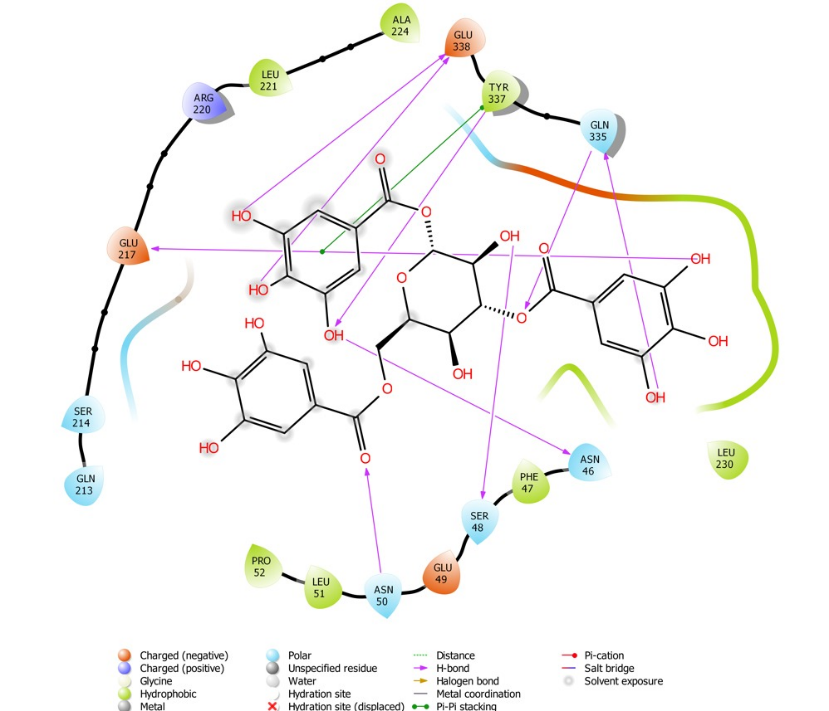

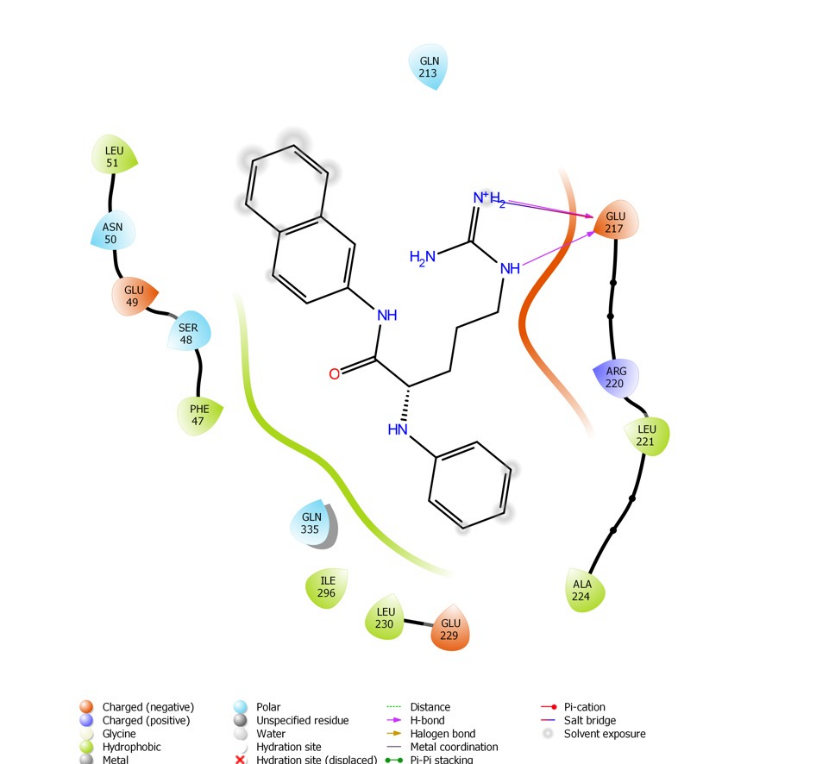


**A B**

**Figure S3:** **LigPlot shows that Gallotannin relative to PAβN exhibits more favorable interactions with AdeA from *A. baumannii*** A)- AdeA interaction with Gal, B-AdeA interaction with PABN.


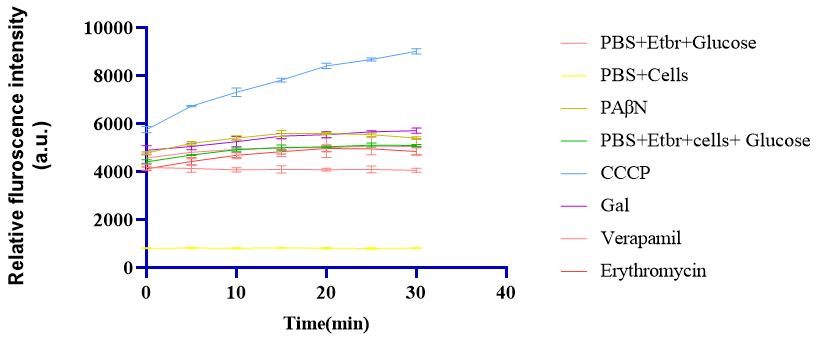


**Figure.S4:** **Gallotannin inhibits EtBr efflux pump in XDR *A. baumanii* BC2267**. The cells of *A. baumanii* BC2267 was treated with Galloctannin (Gal) for 1 h. The cells were then suplimented with glucose and residual fluorescence of EtBr was measured for a time period of 0–20 min. Phenyl arginine beta naphthylamide (PABN), verapamil, carbonyl cyanide m-chlorophenylhydrazine (CCCP) were maintained as positive controls.


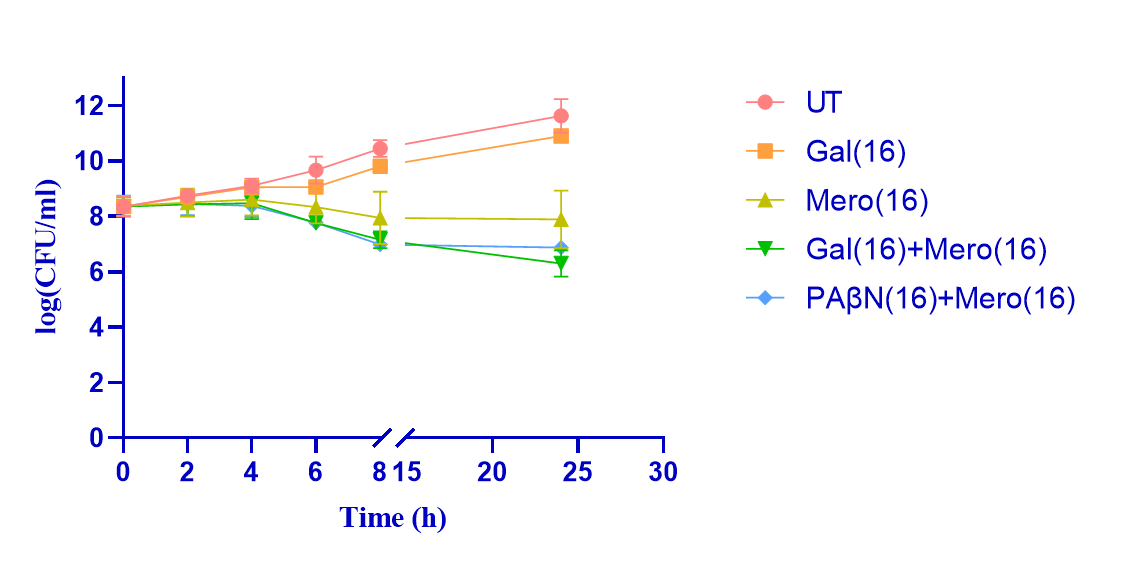
 \

**Figure.S5:** **Gallotannin enhances bactericidal effect of Meropenem in XDR clinical isolate of *A. baumanii* BC2267.** The culture was treated with Gallotannin/Meropenem alone, combination of galloctannin with Meropenem, PABN with Meropenem The samples were withdrawn at time intervals from 0, 2, 4, 6, 8 and 24 h and plated on to LB agar plates, Colony counts were determined and graph was plotted


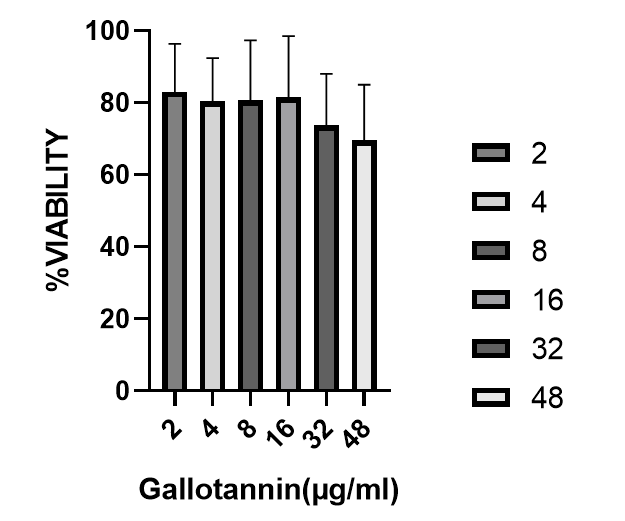


**Figure.S6: Toxicity of Gallotannin evaluated in RAW 264.7 macrophages using MTT assay.** RAW macrophages were grown were seeded into 96-well plates. Following 24 hours of incubation, Gallotannin at various concentrations (2-48µg/ml) were added and cells were incubated further for 24h, after which MTT (0.5 mg/mL) was added and incubated for 1h. The formazan crystals formed by metabolically active cells were dissolved with DMSO and the absorbance of the extracted fraction was measured at 595 nm to assess cell viability.


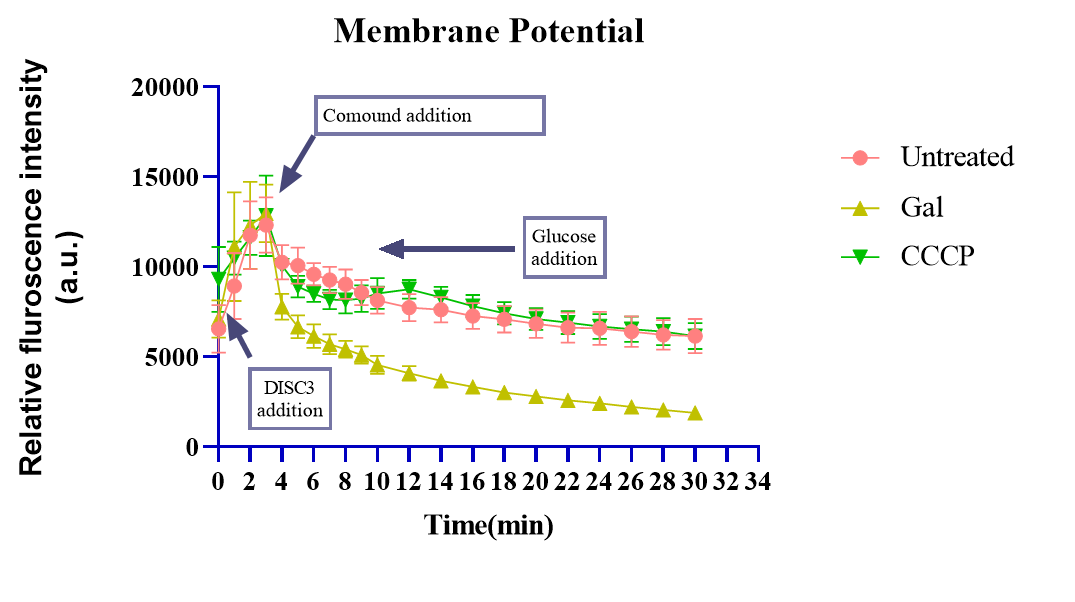


**Figure.S7: Gallotannin did not causes membrane depolarization in XDR clinical isolate of *A. baumanii* BC2267**. Mid log cells *A. baumanii* BC2267 was treated with Gallotannin DiSc3 was used as fluorescent probe, Carbonyl Cyanide m-chlorophenylhydrazine (CCCP) was maintained as positive control. The fluorescent intensity was measured at Ex 605 nm and Em 665 nm.
